# SARS-CoV-2 infection enhances mitochondrial PTP complex activity to perturb cardiac energetics

**DOI:** 10.1016/j.isci.2021.103722

**Published:** 2022-01-01

**Authors:** Karthik Ramachandran, Soumya Maity, Alagar R. Muthukumar, Soundarya Kandala, Dhanendra Tomar, Tarek Mohamed Abd El-Aziz, Cristel Allen, Yuyang Sun, Manigandan Venkatesan, Travis R. Madaris, Kevin Chiem, Rachel Truitt, Neelanjan Vishnu, Gregory Aune, Allen Anderson, Luis Martinez-Sobrido, Wenli Yang, James D. Stockand, Brij B. Singh, Subramanya Srikantan, W. Brian Reeves, Muniswamy Madesh

**Affiliations:** 1 Department of Medicine, Center for Precision Medicine, Cardiology, Infectious Disease Divisions, University of Texas Health San Antonio, San Antonio, TX 78229, USA; 2 Department of Pathology, UT Southwestern Medical Center, Dallas, TX 75390, USA; 3 Department of Internal Medicine, Section on Cardiovascular Medicine, Wake Forest School of Medicine, Winston-Salem, NC 27157 USA; 4 Department of Physiology, University of Texas Health San Antonio, San Antonio, TX 78229, USA; 5 Department of Periodontics, University of Texas Health San Antonio, San Antonio, TX 78229, USA; 6 Texas Biomedical Research Institute, San Antonio, TX 78227, USA; 7 Institute for Regenerative Medicine, University of Pennsylvania, Philadelphia, PA 19104, USA; 8 Department of Pediatrics, Greehey Children's Cancer Research Institute, Division of Hematology-Oncology, University of Texas Health San Antonio, San Antonio, TX 78229, USA; 9 Zoology Department, Faculty of Science, Minia University, El-Minia 61519, Egypt

**Keywords:** Cardiovascular medicine, Virology, Transcriptomics

## Abstract

SARS-CoV-2 is a newly identified coronavirus that causes the respiratory disease called coronavirus disease 2019 (COVID-19). With an urgent need for therapeutics, we lack a full understanding of the molecular basis of SARS-CoV-2-induced cellular damage and disease progression. Here, we conducted transcriptomic analysis of human PBMCs, identified significant changes in mitochondrial, ion channel, and protein quality-control gene products. SARS-CoV-2 proteins selectively target cellular organelle compartments, including the endoplasmic reticulum and mitochondria. M-protein, NSP6, ORF3A, ORF9C, and ORF10 bind to mitochondrial PTP complex components cyclophilin D, SPG-7, ANT, ATP synthase, and a previously undescribed CCDC58 (coiled-coil domain containing protein 58). Knockdown of CCDC58 or mPTP blocker cyclosporin A pretreatment enhances mitochondrial Ca^2+^ retention capacity and bioenergetics. SARS-CoV-2 infection exacerbates cardiomyocyte autophagy and promotes cell death that was suppressed by cyclosporin A treatment. Our findings reveal that SARS-CoV-2 viral proteins suppress cardiomyocyte mitochondrial function that disrupts cardiomyocyte Ca^2+^ cycling and cell viability.

## Highlights

• SARS-CoV-2 perturbs human ion channel, quality control, and mitochondrial transcripts
• SARS-CoV-2 proteins interact with permeability transition pore complex
• SARS-CoV-2 alters VDCC activity that disrupts cardiomyocytes Ca^2+^ cycling
• Cyclosporin A preserves mitochondrial energetics from SARS-CoV-2 infection

## Graphical abstract

**Figure:**
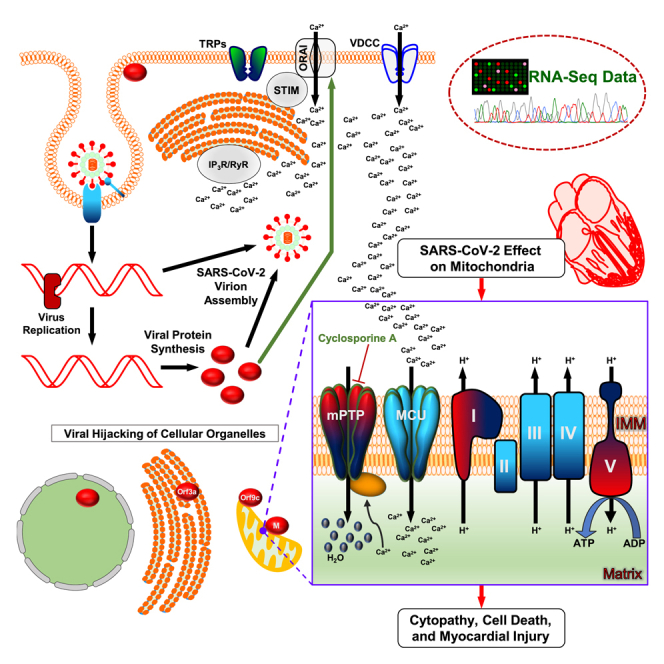

## Introduction

Although several million people worldwide have been infected with coronavirus-SARS-CoV-2, the causative agent of the coronavirus disease 2019 (COVID-19) pandemic, our understanding of the fundamental mechanisms underlying the clinical manifestation of COVID-19 is still emerging. The SARS-CoV-2 entry receptor, ACE2 is highly expressed in several cell types, including myocardial cells, kidney proximal tubule cells, type II alveolar cells, and lymphocytes located in oral mucosa and other tissues. Beyond this canonical entry pathway, the mechanisms of cellular damage and tissue injury are poorly understood but are of paramount importance. Although the virus commonly causes respiratory distress, ≈33% of patients exhibit clinical or laboratory evidence of myocardial injury. Myocardial injury is especially common in COVID-19 patients with a history of cardiovascular disease (30%–60%). A deeper understanding of the mechanisms underlying myocardial injury is needed to develop a therapeutic strategy for such patients with co-morbidities.

Diverse viruses, including influenza M2, HIV-1, and coronavirus E, can undermine the cellular ion homeostasis machinery to allow viral entry and replication, either by co-opting the host ion channels' ability to maintain membrane potential (Fujioka et al., 2018; Hover et al., 2017) or by forming ion channels of virally encoded short membrane proteins that oligomerize to form hydrophilic pores that facilitate ion transport across host cell membranes and override the cellular homeostatic circuits (Wang et al., 2011). However, it is unknown whether SARS-CoV-2 adopts similar mechanistic routes. Among ion channels, the host Ca^2+^ channels influence every process of viral infection and viruses usurp host Ca^2+^ channels to disturb the homeostatic balance in such a way to benefit the virus life cycle at the cost of host survival. For example, influenza A virus infection is initiated by the interaction of the viral glycoprotein hemagglutinin with the voltage-dependent Ca^2+^ channel Ca_v_1.2 to trigger intracellular Ca^2+^ oscillations and subsequent viral entry and replication (Fujioka et al., 2018). Of importance, in cardiac muscle, Ca^2+^ influx is also mediated through the L-type Ca^2+^ channel, suggesting that alterations of voltage-gated calcium channels could be an important factor in COVID-19 and myocardial injury. Inhibition of store-operated Ca^2+^ channel activity reduced the viral egress, and thus infectivity, of many enveloped RNA viruses (Chen et al., 2019; Han et al., 2015). This is evidenced by recent repurposing of existing drugs to treat SARS-CoV-2 infection (Gordon et al., 2020). Specifically, pharmacological agents targeting Sigma1 receptors, known to activate IP_3_R-dependent Ca^2+^ efflux (via store-operated channels) from the ER (Gordon et al., 2020), showed activity against SARS-CoV-2 infection. We have recently reported that HIV-1 Nef-mediated inhibition of cellular protein quality control is one possible mechanism involved in the development of HIV-associated cardiomyopathy and progression toward heart failure (Cheung et al., 2019; Gupta et al., 2017; Muthumani et al., 2006; Natarajaseenivasan et al., 2018; Tahrir et al., 2018). Our recent molecular characterization of the mitochondrial permeability transition pore (PTP) revealed that viral proteins, bacterial toxins, and ion dysregulation elicit PTP and induce cardiac cell death (Shanmughapriya et al., 2015b). Interesting, a new report revealed that SPG7 is upregulated upon SARS-CoV-2 infection (Bojkova et al., 2020), suggesting a role of PTP-mediated mitochondrial dysfunction in determining COVID-19 development and associated myocardial injury.

Although much work has focused on innate and adaptive immune dysregulation in the pathophysiology of COVID-19, the effect of SARS-CoV-2 infection on cellular metabolic activity is unknown. Here we deliver findings that reveal how viral proteins hijack intracellular organelles, ion channels, bioenergetics, and quality control. Our unbiased RNA-seq analysis of uninfected individuals, patients who tested positive for SARS-CoV-2 but were asymptomatic, and severely affected COVID-19 patients reveal numerous human transcripts that were either upregulated or suppressed in the severely affected COVID-19 patients when compared with counterparts. Intriguingly, mitochondrial DNA-encoded transcripts were markedly suppressed, whereas nuclear-encoded mitochondrial respiratory complex, mitochondrial permeability transition pore (mPTP), and mitophagy-related transcripts were markedly elevated in severely affected COVID-19 patients. These severely affected COVID-19 patients showed elevated levels of troponin-C and IL-6 in the plasma. Having observed the elevated cardiac injury marker in COVID-19 samples, we utilized iPSC-derived human cardiomyocytes (iPSC-CMs) to explore the host molecular pathways targeted by SARS-CoV-2 viral proteins. Although SARS-CoV-2 encodes 28 gene products, our comprehensive targeted analyses demonstrate that the viral structural protein, Membrane (M), along with NSP6, NSP10, ORF3a, and ORF9c proteins, discretely localize to mitochondria, endoplasmic reticulum (ER), or plasma membrane compartments. Reconstitution of M-protein in iPSC-CMs markedly inhibited Ca_V_1.2 channel currents. Moreover, ectopic expression of M-protein in human cells resulted in mitochondrial swelling, possibly through the strong interaction with mitochondrial permeability transition pore components SPG7 and CCDC58 to enhance pore opening and causing mitochondrial dysfunction.

## Results

### Unbiased RNA profiling exhibits perturbation of mitochondrial energetics, ion dynamics, and cellular quality control upon SARS-CoV-2 infection in humans

**Figure 1:**
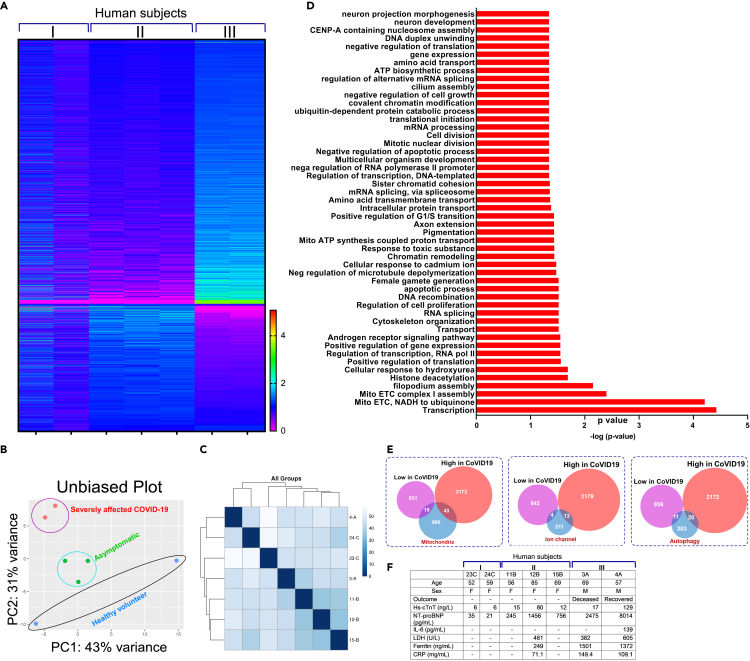
Transcriptome profiling reveals robust perturbation of mitochondrial energetics components during COVID-19 progression (A) Heatmap of all RNA transcripts in PBMCs from healthy SARS-CoV-2-negative volunteers (n = 2), symptomatic (n = 3), and severely affected COVID-19 patients (n = 2). (B) PCA plot of normalized FPKM of RNA-seq from seven samples. (C) Unsupervised hierarchical cluster analysis of COVID-19 patient transcripts. (D) List of enriched transcript categories differentially expressed in COVID-19 patients. (E) Venn diagram represents three major categories of gene ontology. (F) Patient characteristics and blood test results.

To understand the global transcriptional regulation of human mRNAs upon SARS-CoV-2 infection, we utilized PBMCs obtained from (1) uninfected individuals, (2) SARS-CoV-2-positive patients with mild symptoms, and (3) SARS-CoV-2-positive patients who were admitted to the intensive care unit at the University of Texas Southwestern Dallas (UTSW) (IRB Protocol number STU-2020-0366) (Table S1). The RBC contamination in the blood samples was eliminated by routine RBC lysis protocol followed by PBMC purification and total RNA isolation. Total RNA was isolated using the BSL-3 facility at Texas Biomedical Research Institute (TBRI) followed by RNA quantification. Total RNA isolated from PBMCs was depleted of rRNA and globin and used for library construction (NEBNext Ultra RNA Library Prep Kit). The sequencing libraries were multiplexed and sequenced on the Illumina HiSeq instrument using a 2 × 150 paired-end configuration. Image analysis and base calling are conducted by the HiSeq Control Software. Raw sequence data (.bcl files) generated from Illumina HiSeq were converted into fastq files and de-multiplexed using Illumina's bcl2fastq 2.17 software. One mismatch is allowed for index sequence identification. Using DESeq2, a comparison of gene expression between the groups of samples was performed at GENEWIZ (GENEWIZ, South Plainfield, NJ). Using these comprehensive data, we generated a heatmap that depicts the segregation of elevated versus downregulated RNA transcripts of PBMCs (Figure 1A). A principal component analysis (PCA) was performed using the "plot PCA" function within the DESeq2 R package (Data S1). Although the sample size was small, the dynamism of RNA clustering is tight in SARS-CoV-2 infected samples indicating that host cellular transcriptome changes were controlled by the virus (Figure 1B). These RNA transcripts were assessed, and distribution of individual subjects were plotted (Figure 1C). The GO analysis was used to functionally cluster the set of genes based on their biological process and determine their statistical significance (Figure 1D; Data S1). Although our data included both sexes, the severely affected human subjects were male, which possibly could have an influence on the differential gene expression among groups (Table S1 and Data S1). Using this computational analysis, we focused on the highly significantly altered RNA transcripts induced upon viral infection. We found that mitochondrial energetic complex components, ion channel subunits, and autophagy and mitophagy-regulated translational precursors were markedly altered and thus focused our studies on these (Figure 1E). Intriguing, the severely affected COVID-19 patient samples showed elevated cardiac injury markers such as troponin-C, proBNP, and LDH, suggesting acute cardiac damage manifestation in these patients (Figure 1F).

### SARS-CoV-2 viral proteins localized in mitochondria, ER, and plasma membrane, which modulates organelle phenotype

**Figure 2:**
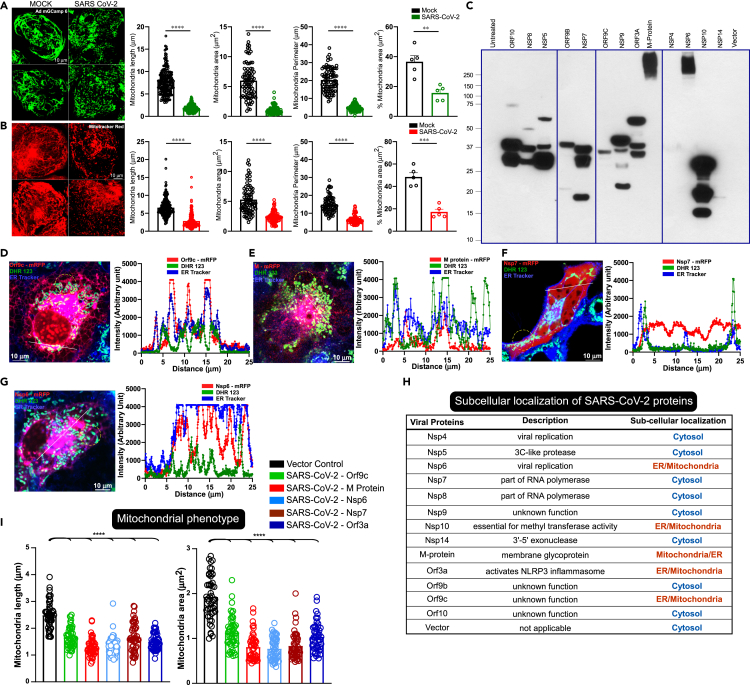
SARS-CoV-2 infection and its proteins targeting promote mitochondrial fragmentation in human iPSC-cardiomyocytes (A) Representative confocal images of Ad mitoGCaMP6 as a mitochondrial marker in iPSC-CMs before or after 24 h of SARS-CoV-2 infection. SARS-CoV-2 MOI 1. Bar graphs depict the analysis of mitochondrial shape parameters. (B) Representative confocal images of MitoTracker Red as a mitochondrial marker in iPSC-CMs before or after 24 h of SARS-CoV-2 infection. SARS-CoV-2 MOI 1. Bar graphs depict the analysis of mitochondrial shape parameters. (A and B) Data are presented as the mean ± SEM, n = 5 independent experiments. ∗∗p <0.01, ∗∗∗p <0.01, ∗∗∗∗p <0.0001. (C) Ectopic expression of individual SARS-CoV-2 proteins in COS-7 Cells. (D-H) Individual viral proteins were transiently transfected in COS-7 cells to visualize the intracellular localization of viral proteins tagged with mRFP (red) on a single-cell basis. Representative confocal images of live cells stained with ER (ER Tracker, blue) and mitochondrial markers (DHR123, green). Spatial overlap and intensity profiles demonstrate ER and mitochondrial localization of viral proteins. (H) Table depicts subcellular localization of thirteen SARS-CoV-2 proteins. (I) Analysis of mitochondrial phenotypes (length and area) in cells expressing SARS-CoV-2 proteins. Data are presented as mean ± SEM, n = 3–6 independent experiments. ∗∗∗∗p <0.0001.

Having observed a cardiac stress marker elevation in severely affected COVID-19 patients, we next tested whether SARS-CoV-2 infection causes cardiomyocyte damage. We utilized human-iPSC-derived cardiomyocytes as a surrogate model system to determine the severity of SARS-CoV-2 infection. To determine the mitochondrial changes, we expressed Ad-mito-GCamp6 or stained with MitoTracker Red as mitochondrial markers. Upon SARS-CoV-2 infection, we found that mitochondrial shape change and fragmentation were evident in virus-infected cardiomyocytes indicating that the viral proteins possibly target mitochondria and impact bioenergetics (Figures 2A and 2B). Based on prediction algorithms and proteomic analysis (Gordon et al., 2020), we cloned and expressed mRFP/FLAG-tagged 13 of the 29 SARS-CoV-2 proteins in COS-7 cells to monitor their cellular distribution and localization. Since these individual candidate genes were tagged with FLAG, we transiently transfected them in COS-7 cells and high-level ectopic protein expression was confirmed by western blotting (Figure 2C). High-resolution imaging of the SARS-CoV-2 proteins was performed using the mRFP-tag in live cells loaded with ER tracker and mitochondrial marker (DHR123) to determine the spatial overlap and intensity profiles (Figure S1). The imaging results show that four of these proteins, Orf9C, M-protein, Nsp6, and Orf3a, were localized to either ER or mitochondrial membranes (Figures S1A, S1B and 2D–2H). In particular, M-protein was predominantly localized in the mitochondrial compartment (Figures 2D and 2H) Remarkably, Nsp7 exhibited ER remodeling and transmigrated near the plasma membrane (Figure 2F). While assessing their host cellular distribution, we observed that M-protein expression induced mitochondrial morphological changes (Figures 2D and 2I). These results indicate that the viral replication and structural SARS-CoV-2 proteins targeted to host cellular organelles besides their canonical function.

### SARS-CoV-2 virus profoundly affects host mitochondrial bioenergetic complexes

**Figure 3:**
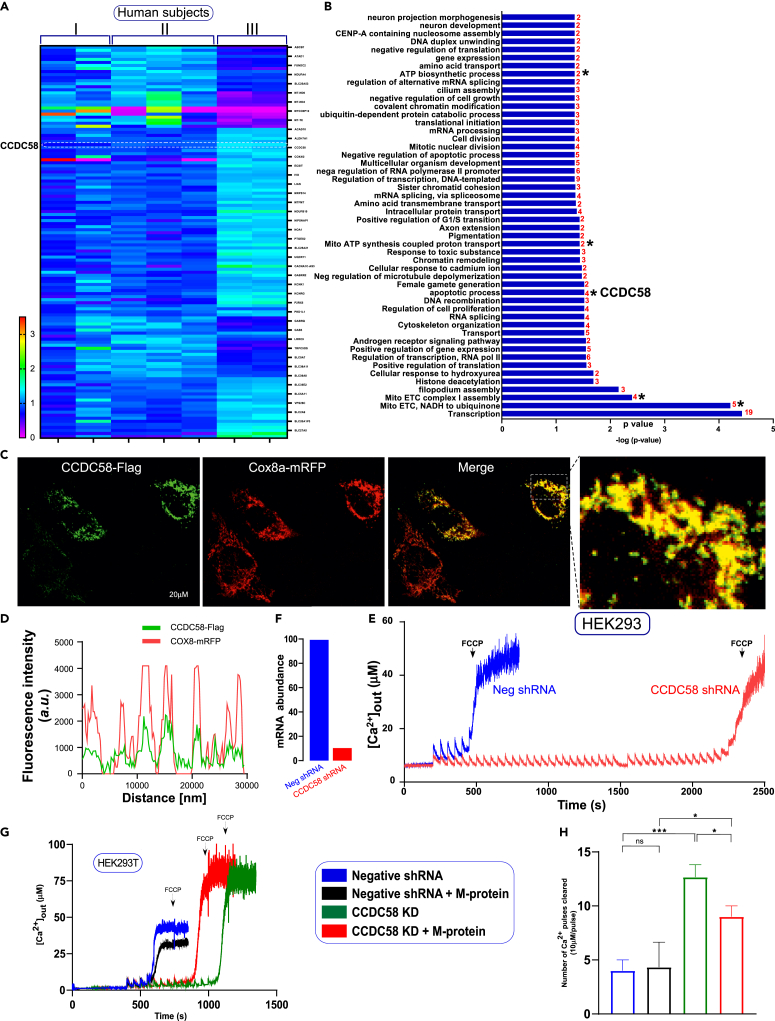
Severely affected COVID-19 PBMCs exhibited an elevation of mitochondrial resident CCDC58 candidate (A-B) Heatmap and plot of mitochondrial perturbation subpopulations from groups I, II, and III human subjects. (C) Assessment of CCDC58 cellular localization. HeLa cells were transiently cotransfected with FLAG-tagged CCDC58 and mitochondrial marker Cox-8A-mRFP plasmid constructs. Immunofluorescence analysis of CCDC58 localization shows the mitochondrial localization. (D) Spatial overlap and intensity profiles demonstrate mitochondrial colocalization of CCDC58 and COX8A mitochondrial targeting polypeptide. (E) Representative traces of Ca^2+^ uptake in NegshRNA or CCDC58 KD HEK293 cells. (F) CCDC58 mRNA expression in NegshRNA or CCDC58 shRNA HEK293 cells. Mean ± SEM. n = 3. (G) Representative traces of Ca^2+^ uptake in HEK293T cells expressing Neg shRNA, NegshRNA + M-Protein, CCDC58 KD, or CCDC58 KD + M-Protein. (H) Calcium retention capacity in HEK293T cells quantified as the number of Ca^2+^ (10 μM) pulses taken up. Data presented as mean ± SEM. n = 3. ∗p <0.05 ∗∗∗p <0.001

The RNA profiling analysis showed that both nuclear and mitochondrial genome encoded transcripts were markedly altered in PBMCs from severely affected COVID-19 patients (Figure 3A; Data S1). Since severely affected COVID-19 patient outcomes include organ failure and death, we focused on candidate genes that promote cell death and mitochondrial dysfunction. Of these transcripts, the previously uncharacterized CCDC58 was significantly elevated in PBMCs obtained from COVID-19 patients (Figure 3B). Based on the mitoCarta and Mito-miner database, CCDC58 is predicted to target mitochondrial compartment (Pagliarini et al., 2008). To assess its localization, C-terminal FLAG-tagged CCDC58 was co-transfected with the mitochondrial marker protein COX8A-mRFP. As predicted, immunofluorescence analysis confirmed that CCDC58 localized predominantly to mitochondria and exhibited near complete overlap with COX8A (Figures 3C, 3D, S2A, and S2B). It has been reported that high-throughput RNAi-mediated silencing of mitochondrial resident proteins, including CCDC58, results in increased mitochondrial Ca^2+^ retention capacity suggesting a link between mitochondrial Ca^2+^ overload and cell death (Shanmughapriya et al., 2015a). Therefore, we determined if stable knockdown of CCDC58 produces resistance to Ca^2+^-induced mitochondrial permeability transition pore opening. The stable transfected control (NegshRNA) and CCDC58 KD 293T cells were subjected to permeabilization using digitonin containing intracellular medium. Upon exposure to excessive extramitochondrial Ca^2+^, control cells exhibited rapid mitochondrial membrane potential (ΔΨ_m_) loss due to Ca^2+^-induced PTP opening (Figures 3E and S3). In contrast, knockdown of CCDC58 markedly increased mitochondrial Ca^2+^ retention capacity (Figures 3D and 3F). In addition, CCDC58 KD cells exhibited higher oxygen consumption rate (OCR) but cellular ATP levels were lowered (Figures S4A–S4H). CCDC58 KD cells exposed to oxidants still partially retained Calcein indicating that CCDC58 likely participates in the mPTP complex (Figures S4I–S4J). Having observed an elevation of CCDC58 under COVID-19 disease development, we next asked whether SARS-CoV-2 M-protein targets CCDC58 protein to alter mitochondrial function. To test this, we reconstituted M-protein in CCDC58 KD cells and monitored CRC. Remarkably, cells lacking CCDC58 were able to maintain CRC when compared with control cells, highlighting a critical role for CCDC58 in COVID-19-induced mitochondrial dysfunction (Figures 3G and 3H).

### SARS-CoV-2 proteins interact with mPTP complex components SPG7, ANT, ATP synthase, CypD, and CCDC58

**Figure 4:**
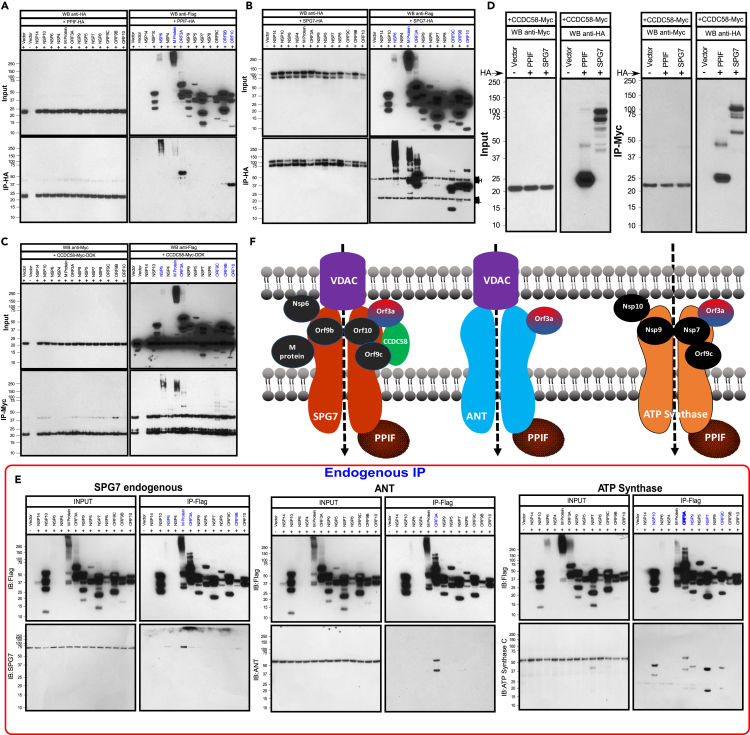
SARS-CoV-2 proteins interact with mitochondrial PTP complex (A) COS-7 cells were cotransfected with HA-tagged PPIF and FLAG-tagged SARS-CoV-2 protein plasmid constructs. Following immunoprecipitation with HA antibody, total cell lysates and immunoprecipitated materials were subjected to western blot analysis. Cell lysates were probed with anti-FLAG or anti-HA antibodies. Immunoprecipitated samples were probed with anti-FLAG (top right) and anti-HA antibodies (bottom right). n = 3. (B) Western blot analysis of cell lysates (left) or immunoprecipitates (right) from COS-7 cells coexpressing HA-tagged SPG7 and FLAG-tagged SARS-CoV-2 protein plasmid constructs. n = 3. (C) Western blot analysis of cell lysates (left) or immunoprecipitates (right) from COS-7 cells coexpressing Myc-tagged CCDC58 and FLAG-tagged SARS-CoV-2 protein plasmid constructs. n = 3. (D) Western blots of cell lysates or immunoprecipitated material from COS-7 cells transiently coexpressing PPIF, SPG7, or CCDC58 proteins. Cells were lysed, immunoprecipitated with Myc antibody, and immunoblotted for HA. n = 3. (E) SARS-CoV-2 proteins and Mitochondrial PTP Complex binding. COS-7 cells were transfected with FLAG-tagged SARS-CoV-2 protein plasmid constructs. Following immunoprecipitation with FLAG antibody, SPG7, ANT, and ATP synthase interactions were assessed by western blot using specific antibodies. (F) Scheme depicts protein-protein interaction of mitochondrial PTP complex with SARS-CoV-2 proteins.

Having observed a robust elevation of CCDC58 and Ca^2+^ dependent functional changes of mitochondrial energetics in COVID-19 patients, we sought to identify SARS-CoV-2 proteins that bind to SPG7 in the mitochondria. We utilized C-terminal hemagglutinin (HA)-tagged cyclophilin D (PPIF) or SPG7 and FLAG-tagged 13 genes of SARS-CoV-2. Transfection of COS-7 cells with these tagged proteins showed substantial ectopic expression (Figures 4A, 4B and S5A). These individual genes were transiently co-transfected with cyclophilin D or SPG-7 in COS-7 cells, and cell lysates were subjected to immunopurification with HA antibody to identify the protein complexes. HA-tagged SPG-7 was immunoprecipitated with NSP6, M-protein, ORF3a, ORF9b, ORF9c, and ORF10 (Figures 4A, 4B and S5A), whereas cyclophilin D interacts with NSP6, M-protein, ORF3A, ORF9b, and ORF10 (Figure 4A). Next, we used CCDC58 as a bait. C-terminal Myc-tagged CCDC58 was cotransfected with the FLAG-tagged 13 viral proteins in COS-7 cells (Figure 4C). Remarkably, NSP6, M-protein, ORF3A, ORF9c, ORF9b, and ORF10 were pulled down using an antibody specific for Myc (CCDC58) (Figure 4C). We have also confirmed these interactions by reverse IP as well as endogenous mPTP complex interactions (Figures S5B and S5C). Since knockdown of CCDC58 resulted in increased mitochondrial Ca^2+^ retention capacity, we next tested whether PPIF and SPG7 interact with CCDC58. COS-7 cells were cotransfected with either CCDC58/PPIF or CCDC58/SPG7 plasmids (Figures 4D and S5A). Remarkably, ectopic reconstitution of CCDC58 in COS-7 cells showed a strong interaction with SPG7 and PPIF indicating that CCDC58 interacts with SPG7 due to its localization in the mitochondrial intermembrane space (Figures 4D and S5A). In addition, we have examined the SARS-CoV-2 proteins interaction with ANT and ATP synthase (Alavian et al., 2014; Bonora et al., 2013; Giorgio et al., 2013; Karch et al., 2019; Shanmughapriya et al., 2015a; Zhou et al., 2019). SARS-Cov-2 plasmids were ectopically expressed in COS-7 cells and immunoprecipitated for SPG7, ANT, ATP synthase binding (Figure 4E). Invariably, SARS-CoV-2 proteins interact with human mPTP complex components (Figure 4E). Collectively, these results indicate that SARS-CoV-2 proteins target multiple organelles, particularly mitochondria, which selectively interacts with mPTP complex components (Figure 4F).

### SARS-CoV-2 infection promotes ER remodeling and exacerbates autophagy in cardiomyocytes

**Figure 5:**
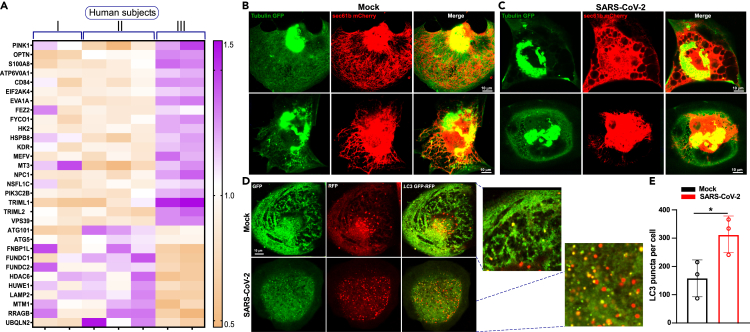
SARS-CoV-2 infection exacerbates autophagy in human iPSC-cardiomyocytes (A) Heatmap depicts the enrichment of cellular and mitochondrial quality control transcripts in severely affected COVID-19 patients. (B and C) Effect of SARS-CoV-2 on cardiomyocytes cyto-skeletal and ER architecture. Representative confocal images of Tubulin-GFP and ER marker (Sec61b-mCherry) expressing iPSC-CMs before or after 24 h of SARS-CoV-2 infection. A multiplicity of infection (MOI) of 1 was used for SARS-CoV-2 in Ad5-tfLC3 (mRFP-GFP tandem fluorescent-tagged LC3, tfLC2, adenovirus)-infected iPSC-CMs. (D) Representative confocal images of mRFP-GFP tfLC3 in iPSC-CMs before or after 24 h of SARS-CoV-2 infection. SARS-CoV-2 MOI 1. (E) Quantification of autophagy was performed as normalized LC3 puncta. Data are presented as the mean ± SEM, n = 3–4 independent experiments. ∗p <0.05.

We again performed a reductionist approach to determine whether these mitochondrial functional changes result in aberrant organelle quality control upon SARS-CoV-2 infection. Our targeted transcriptomic analysis showed that autophagy/mitophagy markers were significantly elevated in PBMCs derived from severely affected COVID-19 patients (Figure 5A; Data S1). We next asked whether SARS-CoV-2 viral infection reorganizes cytoskeletal apparatus and ER network in human-iPSC-derived cardiomyocytes. Cardiomyocytes were ectopically coexpressed in tubulin-GFP and Sec61b-mCherry adenoviral constructs (AdV5a), and the architecture was visualized using confocal imaging. As expected, myocytes displayed choreographed cytoskeletal and ER compartments (Figure 5B). Upon SARS-CoV-2 infection for 24 h, both cytoskeletal and ER reticular network were collapsed (Figure 5C). Having observed that the live SARS-CoV-2 virus causes cellular stress, we next assessed the effect of the virus on the cellular quality control mechanism. Autophagy is a dynamic phenomenon involving autophagosome formation, autophagosome/lysosome fusion, and clearance by lysosomes. To assess whether autophagy was impacted by viral infection, we monitored autophagic flux in cardiomyocytes after SARS-CoV-2 infection using adenovirus expressing a tandem LC3 (light chain 3)-GFP (green fluorescent protein)-RFP (red fluorescent protein) reporter that enables the detection of the autophagic flux process (Figure 5D). Green puncta indicate autophagosome formation, whereas red puncta indicate autophagolysosome formation. In the acidic environment of lysosomes, the GFP signal is quenched. Under basal culture conditions, we observed nominal levels of LC3 puncta in both compartments, whereas cardiomyocytes infected with SARS-CoV-2 for 24 h elicited massive number of positive autophagic vesicles (Figures 5D and 5E). These data suggest that viral infection exacerbates autophagic flux that possibly eliminates a large number of dysfunctional mitochondria.

### mPTP blocker cyclosporin A restores mitochondrial Ca^2+^ retention capacity and cardiomyocyte viability after viral infection

**Figure 6:**
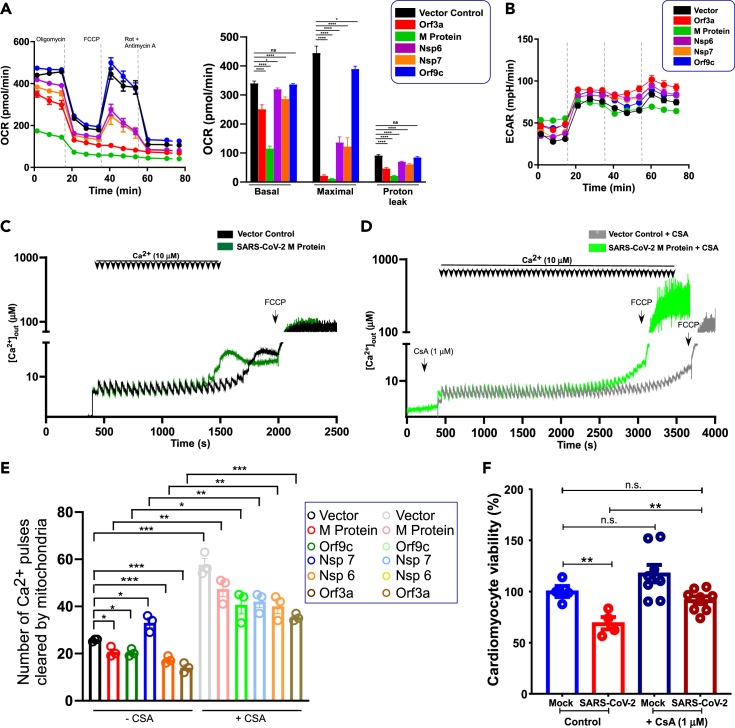
mPTP complex blocker cyclosporin A restores mitochondrial bioenergetics cardiomyocyte viability from SARS-CoV-2-induced damage (A) Mitochondrial oxygen consumption rate (OCR) of vector or SARS-CoV-2 stably expressing COS-7 cells. Bar graph depicts basal, maximal, and proton leak. Mean ± SEM. n = 3–4. The p values were determined by one-way ANOVA with Tukey's test. Data are presented as the mean ± SEM, n = 3–4 independent experiments. ∗p <0.05, ∗∗∗∗p <0001. n.s., not significant. (B) The traces represent extracellular acidification rate and these data are derived from (A) (C-D) Representative traces of number of Ca^2+^ pulses cleared by mitochondria (CRC). Vector or SARS-CoV-2 M-protein stably expressing HEK293 cells were permeabilized and exposed to boluses of 10 μM Ca^2+^ pulses with (C) or without (D) cyclosporin A (1 μM) at the indicated time point. (E) Quantification of mitochondrial CRC in both control and viral protein expressing conditions. Data are presented as the mean ± SEM, n = 3–6 independent experiments. ∗p <0.05, ∗∗p <0.01, ∗∗∗p <001. n.s., not significant. (F) Assessment of cardiomyocyte viability following SARS-CoV-2 infection. hiPSC-cardiomyocytes were challenged with virus at an MOI of 1 for 24 h. After viral infections, cells were subjected to MTT assay to determine the viability. Data are presented as the mean ± SEM, n = 4–8 independent experiments. ∗∗p <0.01, n.s., not significant.

We next tested whether viral proteins alter mitochondrial bioenergetics and possibly induce cardiomyocyte cell death. The mitochondrial oxygen consumption rate (OCR) in HEK293 cells overexpressing viral proteins was measured. Remarkably, cells expressing M-protein or ORF3a had lower basal OCR and minimal FCCP-induced maximal OCR (Figure 6A). Interestingly, NSP6 and NSP7 exhibited a partial suppression of maximal OCR (Figure 6A). However, the ECAR (extracellular acidification rate) was similar among these cell types (Figure 6B). Although OCR was affected by these viral proteins, the cells were viable, indicating a switch from oxidative to glycolytic phenotypes. To further investigate the impact of viral proteins on mitochondrial ion homeostasis, we measured MCU channel activity as well as mitochondrial CRC in the permeabilized cell system. We found that the CRC is reduced by ectopic expression of SARS-CoV-2 proteins; however, the MCU activity remains intact (Figures 6C–6E and S6). We next evaluated whether mPTP blocker cyclosporin A restores the CRC in viral protein expressing condition. As expected, CsA enhanced the mitochondrial CRC but the viral M-protein-mediated CRC reduction was still observed (Figures 6C, 6D and S6). Reconstitution of these viral proteins exhibited lower CRC with the exception of NSP7 (Figure 6E). Finally, we assessed the cardiomyocyte viability under viral infection. Twenty-four hours post infection, cardiomyocyte viability was assessed using MTT assay (Figure 6F). Cardiomyocytes exposed to virus exhibited a significant cell death that was considerably prevented by cyclosporin A treatment suggesting that these viral particle/proteins could disrupt mitochondrial bioenergetics through mPTP opening and eventual cardiomyocyte damage (Figure 6F).

### SARS-CoV-2 proteins disrupt cardiomyocyte Ca^2+^ cycling and suppress voltage-gated Ca_V_1.2 Ca^2+^ channel activity

**Figure 7:**
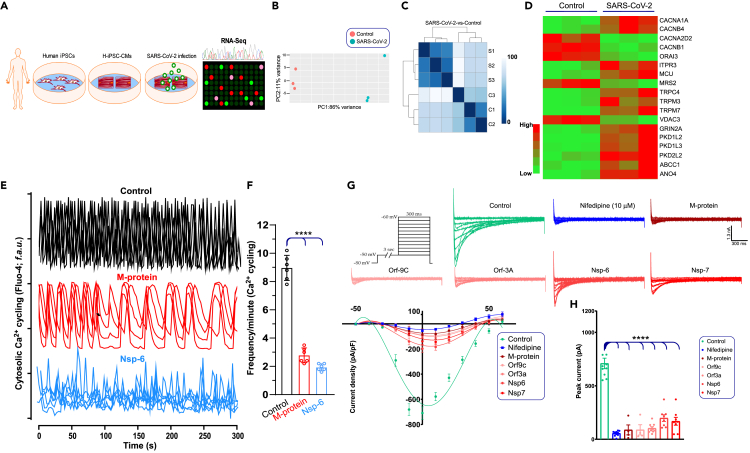
SARS-CoV-2 proteins suppress LTCC channel activity in cardiomyocytes (A) Schematic overview of the study. Twenty days after the start of differentiation, hiPSC-CMs were exposed to SARS-CoV-2 virus (1 MOI) for 24 h before RNA isolation to perform RNA-seq analysis. (B) PCA plot of normalized FPKM of RNA-seq from control (n = 3) and SARS-CoV-2-infected hiPSC-CMs (n = 3). (C) Cluster analysis of control and SARS-CoV-2 infected hiPSC-CMs. (D) Targeted ion channel transcripts differentially modulated in hiPSC-CMs upon SARS-CoV-2 infection (n = 3). (E) Representative optical recordings show Fluo-4 Ca^2+^ cycling traces in both control (RFP-tagged plasmid) and RFP-tagged SARS-CoV-2 M-protein or NSP-6 overexpressing hiPSC-CMs. (F) Quantification of Ca^2+^ oscillations frequency in cardiomyocytes. These data are derived from traces in A. Data are presented as the mean + SEM, n = 3–4 independent experiments. (G) Current-voltage relationship of Ca_V_1.2 channels in control and viral protein-overexpressing hiPSC-CMs. (H) Peak current densities (pA) for control or SARS-CoV-2 expressing hiPSC-CMs. n = 6–12. Data are presented as the mean ± SEM, n = 4–10 independent experiments. ∗∗∗∗p <0.0001.

The RNA-seq analysis from the severely affected patients revealed a significant elevation of Ca^2+^ channel component transcripts, in particular, LTCC (CACNA1C) and the CRAC channel regulator, STIM1, were upregulated (Figure 1). Having shown that SARS-CoV2 infection alters the expression of ion channels in cardiomyocytes, we next determined the impact of SARS-CoV-2 on cardiomyocyte function using hiPSC-CMs. We first determined the effect of SARS-CoV-2 infection on RNA transcript changes in human iPSC derived cardiomyocytes (hiPSC-CMs). hiPSCs-CMs were developed as described previously (Shanmughapriya et al., 2018) and infected with SARS-CoV-2 virus for 24 h. The targeted RNA-seq analysis exhibited a significant alteration of ion channels transcripts; in particular, Ca^2+^ channels (LTCC subunits, TRP, IP_3_R, and MCU) were elevated in SARS-CoV-2-infected hiPSC-CMs (Figures 7A–7D; Data S2 and Data S3) (Baughman et al., 2011; Csordas et al., 2013; De Stefani et al., 2011; Dong et al., 2017; Kirichok et al., 2004; Mallilankaraman et al., 2012a, 2012b; Pan et al., 2013). Conversely, the CRAC channel, Orai3, and mitochondrial Mg^2+^ uptake channel Mrs2 were suppressed (Figure 7D) (Daw et al., 2020). Since efficient Ca^2+^ cycling is essential for cardiomyocyte contraction and relaxation, we first tested the spontaneous Ca^2+^ cycling in hiPSC-CMs. A robust Ca^2+^ cycling was observed in control hiPSC-CMs, but this rhythmic Ca^2+^ cycling was perturbed in hiPSC-CMs expressing SARS-CoV-2 M-protein (Figure 7E). Although the Ca^2+^ amplitude was similar, the diastolic concentrations and frequencies of systolic Ca^2+^ were markedly prolonged in hiPSC-CMs expressing SARS-CoV-2 M-protein (Figures 7E and 7F). Similarly, overexpression of NSP-6 exhibited dramatic disruption in both amplitude and Ca^2+^ cycling (Figures 7E and 7F) indicating that impairment of cardiomyocytes Ca^2+^ cycling by viral proteins could alter force generation. Functional characterization (using whole-cell configuration at various membrane potential) further showed the presence of L-type Ca^2+^ currents in control hiPSC-CMs (Figure 7G). The hiPSC-CMs displayed current-voltage (I/V) properties and inhibition by nifedipine typical for Ca_v_1.2 channels (LTCC) (Bers, 2002, 2008, 2014; Catterall et al., 2005) (Figure 7G). Having observed a perturbation of Ca^2+^ cycling, we ectopically expressed SARS-CoV-2 M-protein, ORF-9c, ORF-3a, NSP-6, and NSP-7 in hiPSC-CMs. These viral proteins resulted in significant suppression of Ca_v_1.2 currents (Figure 7H). Collectively, these results reveal that SARS-CoV-2 viral proteins disrupt cardiomyocyte Ca^2+^ cycling and ion channel activity.

## Discussion

Numerous studies have shown that a large percentage of individuals diagnosed with COVID-19 manifest evidence of myocardial injury and that these individuals have a higher risk of death (Calvo-Fernández et al., 2020). The SARS-CoV-2 Spike protein binds to its cellular receptor, the ACE2 protein and TMPRSS2, to enter host cells (Hoffmann et al., 2020). ACE2 is highly expressed on cardiomyocytes (Han et al., 2020), and direct infection of cardiomyocytes by SARS-CoV-2 has been documented in patients (Tavazzi et al., 2020; Tian et al., 2020) and in cultured cardiomyocytes (Wong et al., 2020). However, the mechanisms by which the virus alters cardiomyocyte function remain unclear. To better delineate the pathways perturbed during SARS-CoV-2 infection, we performed RNA-seq analysis of PBMCs from COVID-19 patients. Furthermore, we performed detailed molecular and biochemical studies of human-iPSC-derived cardiomyocytes. In addition, we also characterized cellular organelle (ER and mitochondria) morphological changes upon cellular targeting by SARS-CoV-2 viral proteins in different cell model systems.

First, we determined the global transcriptome profile of SARS-CoV-2 infection in PBMCs obtained from healthy volunteers and symptomatic and severely affected COVID-19 patients. Pathways that were significantly altered in COVID-19 patients with severe illness included transcription, mitochondrial oxidative phosphorylation, ATP biosynthesis, ion channels, autophagy, and apoptosis (Figures 1 and 3). Likewise, oxidative phosphorylation, calcium signaling, and apoptosis have been reported by other groups to be altered in COVID-19 PBMCs (Gardinassi et al., 2020; Xiong et al., 2020). This remarkable regulation suggests that SARS-CoV-2 proteins not only are essential for viral replication, assembly, and cell lysis but also potentially target host cellular compartments to affect cell and organ function. Based on predictions of SARS-CoV-2 protein structure (Gordon et al., 2020), we selected 13 viral proteins for further analysis. Of these, we found that several were localized to ER, mitochondria, and/or plasma membrane. We also observed that these viral proteins resulted in dramatic changes in mitochondrial morphology (Figure 2) and mitochondrial function. These findings emphasize that SARS-CoV-2 proteins have a moonlighting role beside their canonical functions.

Having noted transcriptomic changes associated with oxidative phosphorylation and localization of SARS-CoV-2 proteins to mitochondria, we focused our attention on mitochondrial proteins. Of these, we found a significant elevation of a previously uncharacterized nuclear-encoded mitochondrial protein, CCDC58, in COVID-19 PBMCs (Figures 3A and 3B). A recent report also identified an upregulation of mPTP complex components SPG7 and cyclophilin D in Caco-2 cells infected with SARS-Co-V-2 (Bojkova et al., 2020; Shanmughapriya et al., 2015a; Zhou et al., 2019). Therefore, we explored the role of CCDC58 in the mPTP. We confirmed that CCD58 is localized to the mitochondria (Figure 3C) and showed that knockdown of CCDC58 dramatically enhanced the capacity of mitochondria to sequester calcium. Of interest, expression of the SARS-CoV-2 M protein reduced calcium sequestration in the absence of CCDC58. We then performed a systematic assessment of interactions between SARS-CoV-2 proteins and components of the mPTP. Indeed, we found that SPG7, PPIF, and CCDC58 physically interact with each other and that several SARS-CoV-2 viral proteins, including M protein, ORF3a, ORF9b and 9C, ORF10 and NSP6, bind to mPTP complex proteins (Figure 4) suggesting a likely involvement in COVID-19-induced mitochondrial dysfunction and cell death (Figure 7). Since our results suggested that SARS-CoV-2 may alter mitochondrial morphology and function, we measured the effect of specific SARS-CoV-2 proteins on oxidative phosphorylation and mitochondrial calcium sequestration. Several of the proteins resulted in a substantial inhibition of maximal mitochondrial respiration and reduced the capacity of mitochondria to sequester calcium (Figures 6A–6D). Cyclosporin A, which inhibits the mPTP, reversed these effects. These results suggest that SARS-CoV-2 proteins may alter the mPTP making it more prone to opening and triggering subsequent cell death. Consistent with this view, we found that infection of hiPSC-CMs with SARS-CoV-2 decreased cell viability and that this effect was reversed by cyclosporin A (Figure 6F).

Proper Ca^2+^ dynamics are essential to cardiomyocyte function, and any aberrations lead to cardiac pathological conditions (Bers, 2002, 2008, 2014). The ER, mitochondria, and plasma membranes all participate in the orchestration of calcium dynamics. In addition, to the perturbation of mitochondrial calcium sequestration (Figure 6), we noted that several transcripts involved in calcium transport, such as STIM1 and L-type calcium channels, were upregulated in COVID-19 PBMCs. To explore the effects of SARS-CoV-2 on cardiomyocyte calcium dynamics, we performed electrophysiologic studies on hi-PSC-CMs expressing SARS-CoV-2 proteins. The results show that these proteins dramatically disrupt the baseline oscillations of cell calcium and almost completely block L-type calcium channel activity indicating the cardiomyocyte damage and the development of heart failure.

In conclusion, our results show that SARS-CoV-2 proteins localize to mitochondria, bind to the mPTP complex, alter mitochondrial morphology and function, disrupt Ca^2+^ cycling, and inhibit VDCC channel activity, all indicating a perturbation of ion homeostasis and mitochondrial dysfunction (Figure 7). These observations lead us to speculate that COVID-19-induced cardiac damage may be due to mitochondrial dysfunction. Moreover, these comprehensive analyses point toward new drug targets to preserve host cells against SARS-CoV-2-induced cardiac damage. Specifically, our results suggest that the mPTP blocker and immunosuppressive drug cyclosporin A may offer an additional therapeutic option to treat COVID-19 patients.

### Limitations of the study

Although over 200 million people around the world are infected with coronavirus SARS-CoV-2, the causative agent of the COVID-19 pandemic, an understanding of the fundamental mechanisms underlying the clinical manifestation of COVID-19 is still emerging. Because of the nature of its infectivity, we were able to perform critical *in vitro* experiments with SARS-Co-V-2 virus. To understand the molecular nature and host cellular distribution of the viral proteins, we utilized multiple cell models. Although it is considered as a limitation, our mRNA transcriptomics of human PBMCs and cellular and mitochondrial functional data are consistent and provide new insights into how viral protein components target mitochondria that elicits bioenergetic collapse and cardiomyocyte damage.

## STAR★Methods

### Key resources table

**Table:** 

REAGENT or RESOURCE	SOURCE	IDENTIFIER
**Antibodies**		

Monoclonal ANTI-FLAG® M2	Sigma Aldrich, MO, USA	Cat# F3165
Flag, Rabbit monoclonal antibody	Cell Signaling Technology, MA, USA	Cat# 14793
Myc-Tag (71D10) Rabbit monoclonal antibody	Cell Signaling Technology	Cat# 2278S
β-Actin (C4)	Santa Cruz Biotechnology, TX, USA	Cat# SC47778
HA-Tag Rabbit monoclonal antibody	Cell Signaling Technology, MA, USA	Cat#3724S
Goat Anti-Rat IgG (H+L) Secondary Antibody, Biotin	Invitrogen, NY, USA	Cat# 31830
Anti-ATP5G1	Invitrogen, NY, USA	Cat# PA5-68102
Anti-SPG7	Invitrogen, NY, USA	Cat# PA5-87106
Anti-ANT (H-188)	Santa Cruz Biotechnology, TX, USA	Cat# SC- 11433
Mouse Anti-Rabbit IgG Conformation Specific (L27A9) mAb (HRP)	Cell Signaling Technology, MA, USA	Cat# 5127S
Anti-Cyclophilin F antibody	abcam, MA, USA	Cat# ab110324
Anti-Tom20	abcam, MA, USA	Cat# ab56783
Anti-Cytochrome c	Santa Cruz Biotechnology, TX, USA	Cat# sc13156
Monoclonal anti-ATP5A	abcam, MA, USA	Cat# ab14748

**Bacterial and virus strains**		

SARS-CoV-2	Texas BioMed Research Institute (San Antonio, Texas)	
Ad-h-Sec61-mCherry	Vector BioLabs	ADV-222504
Ad-Tubulin GFP	Vector BioLabs	ADV-226814
Ad-mito-GCamp6m		Tomar et al. (2019)
Lenti-ccdc58 shRNA	Sigma/Aldrich	Shanmughapriya et al. (2015a)

**Chemicals, peptides, and recombinant proteins**		

Halt™ Protease and Phosphatase Inhibitor Cocktail	ThermoFisher Scientific, NY, USA	Cat# 78441
NuPAGE™ LDS Sample Buffer (4X)	ThermoFisher Scientific	Cat# NP0007
Restore™ PLUS Western Blot Stripping Buffer	ThermoFisher Scientific	Cat# 46430
Pierce™ 20X TBS Tween™ 20 Buffer	ThermoFisher Scientific	Cat# 28360
MES SDS Running Buffer (20X)	ThermoFisher Scientific	Cat# B0002
SuperSignal™ West Pico PLUS Chemiluminescent Substrate	ThermoFisher Scientific	Cat# 34578
Bis-Tris Polyacrylamide Gel	ThermoFisher Scientific	Cat# WG1402BOX
Bolt^TM^ LDS sample buffer (4X)	ThermoFisher Scientific	Cat# B0007
RIPA Buffer	Abcam, MA, USA	Cat# 156034
Pierce IP Lysis buffer	ThermoFisher Scientific	Cat# 87788
Non-fat dry skimmed milk	Lab Scientific, MA, USA	Cat# M-0841
Dihydrorhodamine 123 (DHR 123)	ThermoFisher Scientific	Cat# D23806
FURA-FF Pentapotassium Salt	Cayman Chemical, MI, USA	Cat# 20415
JC-1	ThermoFisher Scientific	Cat# 65-0851-38
MitoSOX^TM^ Red	ThermoFisher Scientific	Cat# M3008
FCCP	Sigma Aldrich, MO, USA	Cat# C2920
Tetramethylrhodamine, Ethyl Ester, Perchlorate (TMRE)	ThermoFisher Scientific	Cat# T669
Ionomycin from *Streptomyces conglobatus*	Sigma Aldrich, MO, USA	Cat# I9657
Thapsigargin	Thermo Fisher Scientific	Cat# T7458
Digitonin	Sigma Aldrich, MO, USA	Cat# D141
Thiazovivin	Cayman Chemical, MI, USA	Cat# 14245
Gelatin	MilliporeSigma, MA, USA	Cat# ES-006B
Calcium Chloride	Sigma Aldrich, MO, USA	Cat# 2115
Glucose	Sigma Aldrich, MO, USA	Cat# G8644
Sodium Succinate	Sigma Aldrich, MO, USA	Cat# S2378
DMSO	Sigma Aldrich, MO, USA	Cat# D8418
Fetal Bovine Serum, Characterized, Heat Inactivated	Hyclone (GE Life Sciences)	Cat#SH30396.03
DPBS Ca^2+^/Mg^2+^ Free	Gibco (ThermoFisher Scientific)	Cat#14190
Collagen 1 Rat Tail	Gibco (ThermoFisher Scientific)	Cat#A10483
Sodium Pyruvate, 100mM	Gibco (ThermoFisher Scientific)	Cat# 11360
Hanks' Balanced Salt Solution, Ca^2+^/Mg^2+^ Free	Gibco (ThermoFisher Scientific)	Cat# 14175
HEPES (1M)	Gibco (ThermoFisher Scientific)	Cat# 15630
EGTA, OmniPur (powder)	MilliporeSigma, MA, USA	Cat# 4100-50GM
B-27 Supplement	Gibco (ThermoFisher Scientific)	Cat#17504-044
MEM – Non-Essential Amino Acids, 100X	Gibco (ThermoFisher Scientific)	Cat# 11140
Antibiotic-Antimycotic, 100X	Gibco (ThermoFisher Scientific)	Cat# 15240
L-Glutamine, 200mM	Gibco (ThermoFisher Scientific)	Cat# 250030
Mito Tracker Deep red FM	Invitrogen, ThermoFischer Scientific, USA	Cat# M22426
Neutral buffered Formalin	Cole Parmer, ThermScientific IL, USA	Cat# 5701
DMEM High glucose	Hyclone, Cytiva, MA, USA	Cat# SH30243.01
RPMI Medium	Hyclone, Cytiva, MA, USA	Cat# SH30027.01
Seahorse XF DMEM Medium	Agilent, USA	Cat# 103575-100
Trypsin 0.05%	Gibco (ThermoFisher Scientific)	Cat# 25200-056
Trypsin 0.25%	Gibco (ThermoFisher Scientific)	Cat# 25300-054
Adenosine Triphosphate Mg^2+^ salt	Sigma Aldrich, MO, USA	Cat# A9187
Pierce Anti-c-Myc magnetic Beads	ThermoFischer Scientific, USA	Cat# 88842
Pierce Anti-HA magnetic Beads	ThermoFischer Scientific, USA	Cat# 88836
Β-mercaptoethanol	ThermoFischer Scientific, USA	BP176-100
FLAG HA Tandem Affinity Purification Kiet	Sigma Aldrich, MO, USA	Cat# Tp0010
Precision Plus Protein dual color standards	BIORAD, CA, USA	Cat#1610374
Tetrodotoxin Citrate	TOCRIS, MN, USA	Cat#1069
Nifedipine	Sigma Aldrich, MO, USA	Cat# N7634
Cesium chloride	Sigma Aldrich, MO, USA	Cat# C4036

**Critical commercial assays**		

Pierce™ BCA Protein Assay Kit	ThermoFisher Scientific	Cat# 23225
RNeasy Plus Universal Mini Kit	QIAGEN, MD, USA	Cat# 73404
Agilent Seahorse XF Cell Mito Stress Test Kit	Agilent Technologies, CA, USA	Cat# 103015-100
Agilent Seahorse XFe96 FluxPak	Agilent Technologies	Cat# 102416-100
CellTiter 96 Non-radioactive cell proliferation assay	Promega, USA	Cat# G4002
Cell Titer-Glo Luminescence Kit	Promega, USA	Cat# G7570
iBlot PVDF Regular Stacks	Invitrogen, ThermoFischer Scientific, USA	Cat# IB24001
NEB Next Ultra RNA Library Prep Kit for Illumina	New England Biolabs, MA, USA	Cat# E7530L
FuGENE 6 Transfection Reagent	Promega, USA	Cat# E2691

**Experimental models: Cell lines**		

Human: HEK293 Cells	ATCC	Cat# CRL-1573
Human: HEK293T Cells	ATCC	Cat# CRL-3216
*Cercopithecus aethiops*: COS-7 Cells	ATCC	Cat# CRL-1651
Human HeLa Cervical cancer cells	ATCC	Cat# CCL-2
Human: PBMC Primary Cells	UT Southwestern, TX, USA	N/A
Human: iPSC-derived Cardiomyocytes (PENN123i-SV20)		N/A

**Recombinant DNA**		

pVector-RFP	OriGene, MD, USA	
pM Protein-RFP	OriGene, MD, USA	
pOrf9c-RFP	OriGene, MD, USA	
pOrf3a-RFP	OriGene, MD, USA	
pNsp6-RFP	OriGene, MD, USA	
pNsp7-RFP	OriGene, MD, USA	
pOrf10-RFP	OriGene, MD, USA	
pOrf9b-RFP	OriGene, MD, USA	
pNsp5-RFP	OriGene, MD, USA	
pNsp14-RFP	OriGene, MD, USA	
pNsp8-RFP	OriGene, MD, USA	
pNsp9-RFP	OriGene, MD, USA	
pNsp10-RFP	OriGene, MD, USA	
pNsp4-RFP	OriGene, MD, USA	
pGCamp6-mt	Nemani et al. (2018)	N/A
pCox-8 A mRFP	OriGene, MD, USA	RC100126

**Software and algorithms**		

GraphPad Prism version 8	GraphPad Software	v8.0
Canvas 11.0	ACD Systems	v11.0
Agilent Seahorse Wave Desktop Software 2.6.1	Agilent Technologies, CA, USA	https://www.agilent.com/en/products/cell-analysis/software-download-for-wave-desktop
Leica Application Suite X (LAS X)	Leica Microsystems Inc., IL, USA	https://www.leica-microsystems.com/products/microscope-software/p/leica-las-x-ls/
Agilent Seahorse Wave	Agilent Technologies, CA, USA	Wave 2.6.1
Microsoft Office	Microsoft Corporation, WA, USA	2013/2016
Felix GX Software	PTI, Horiba, Canada	Felix Gx, Version 4
Zen 2010	Carl Zeiss Inc, Ob, Germany	Zeiss, LM510
Zen Blue	Carl Zeiss Inc, Ob, Germany	Zeiss LSM 10 META
R	https://www.cran.r-project.org	v3.6.2
DESeq2 (R Package)	Love, et al., 2014	https://github.com/mikelove/DESeq2
Bcl2fastq	Illumina, CA, USA	v2.17
Illustration Toolkit- Pathway	Motifolio, MD, USA	https://www.motifolio.com
Image J	Schneider et al., 2012	https://imagej.nih.gov/ij/

### Resource availability

#### Lead contact

Further information and requests for resources and reagents should be directed to and will be fulfilled by the lead contact, Muniswamy Madesh (muniswamy@uthscsa.edu).

#### Materials availability

This study did not generate new unique reagents.

### Experimental model and subject details

#### Study oversight and data collection

This study was approved by the institutional review board of the University of Texas Southwestern Medical Center (IRB Protocol number STU-2020-0366).

#### SARS-CoV-2 detection among the patient groups

A nasopharyngeal swab of suspected patients was subjected to real-time SARS-CoV-2 assay (m2000 Abbott) to confirm SARS-CoV-2 infection. SARS-CoV-2 infected patients with varying degrees of severity and uninfected patients were included in this study. For comparison, COVID-19 patients were stratified based on symptoms and the degree of medical care required during their respective hospital courses. Three groups of patients were identified: I) healthy volunteers, II) patients exhibiting mild to moderate COVID-19 associated respiratory disease, and III) severely affected patients presenting with critical COVID-19 manifestations requiring intensive medical intervention. The human subjects' details include sex and/or gender, age, and other additional details are provided in the Table S1.

#### RNA isolation and RNA sequencing

RNA was isolated from remnant clinical blood specimens collected from: I) Healthy volunteers, II) patients positive for SARS-CoV-2 with mild to moderate respiratory symptoms, and III) patients positive for SARS-CoV-2 requiring intensive care. Whole blood EDTA samples were centrifuged to remove the top plasma first before total RNA isolation from the remaining cell pellets. RNA-Seq was performed at GENEWIZ® (GENEWIZ, South Plainfield, NJ). Briefly, total RNA was processed for rRNA and globin depletion, quantified, and its integrity was confirmed prior to library construction using the NEBNext Ultra RNA Library Prep Kit (New England Biolabs, Ipswitch, MA). The sequencing libraries were multiplexed and sequenced on the Illumina HiSeq instrument using a 2x150 paired-end (PE) configuration. Image analysis and base calling were conducted by the HiSeq Control Software (HCS). Raw sequence data (.bcl files) generated from Illumina HiSeq were converted into fastq files and de-multiplexed using Illumina's bcl2fastq 2.17 software. One mismatch was allowed for index sequence identification. Using DESeq2, a comparison of gene expression between the groups of samples. Principal component analysis (PCA) was performed using the "plot PCA" function within the DESeq2 R package.

#### Cell culture

##### hiPSC-CMs differentiation and cardiomyocyte markers

The iPSC line PENN123i-SV20 was used for generating hiPSC-CMs as previously described (Shanmughapriya et al., 2018). The derivation of PENN123i-SV20 was approved by the Institutional Review Board of the University of Pennsylvania. hiPSC-CMs were infected with SARS-CoV-2 using a BSL-3 facility for 24 h, and various SARS-CoV-2 proteins (RFP-labeled constructs) were overexpressed using transient transfection methods. RNA was isolated from infected cells, and the expression of SARS-CoV-2 genes was confirmed using RT-PCR, followed by global RNA-Seq analysis.

COS-7 (ATCC® CRL-1651), HeLa, HEK293 (ATCC® CRL-1573) and HEK293T (ATCC® CRL-3216) cells were cultured in high glucose (4.5 g/L) DMEM (Hyclone, #SH30022) supplemented with 110 mg/L sodium pyruvate, 10% (v/v) FBS (Hyclone, #SH30396.03), and 1% (v/v) antibiotic-antimycotic solution. All cells were cultured at 37°C and 5% CO_2_. Trypsin (0.25% for COS-7 cells, 0.05%, for HEK cells) and TrypLE reagents for hiPSCs were used to detach cells from culture plates for experiments.

### Method details

#### Assessment of subcellular localization by immunofluorescence

HeLa cells were plated on 30 mm diameter, number 1.5 thickness coverslips coated with Poly-L-Lysine. Cells were co-transfected with CCDC58-Flag and Cox8a-mRFP using the FuGENE HD transfection reagent as per manufacturer instructions. After 24 h, cells were fixed with 4% paraformaldehyde for 15 min at room temperature (RT). Fixed cells were washed two-times with DPBS and permeabilized with 0.15% Triton X-100 solution for 15 min at RT. Permeabilized cells were blocked with 10% (w/v) bovine serum albumin (BSA) solution for 45 min at RT and subsequently incubated overnight with monoclonal anti-FLAG M2 antibody for the detection of CCDC58. After incubation, cells were washed three times with 10% BSA, and then the secondary anti-mouse Alexa 488 antibody was added for 1 h at RT. After incubation with secondary antibody, cells were washed three times with Tris-buffered saline with 0.05% Tween 20 detergent (TBST) and imaged at LSM 510 META Confocal Laser Scanning Microscope (Carl Zeiss, Inc.) at 488- and 594-nm excitations using a 40x oil objective. Co-localized pixels were quantified by detecting the fluorescence intensity of CCDC58-Flag and Cox8a-mRFP pixels using the line scan in ZEN blue software (Carl Zeiss, Inc.).

#### Ratiometric Ca^2+^ imaging for mitochondrial calcium retention capacity

Ca^2+^ flux analysis for the mitochondrial calcium retention capacity was performed as described earlier (Shanmughapriya et al., 2015a; Tomar et al., 2016). Briefly, HeLa cells were trypsinized and washed in Ca^2+^ and Mg^2+^ free DPBS, and an equal number of cells (6x10^6^ cells) were resuspended and permeabilized with 40 μg/mL digitonin in 1.5 mL of a Ca^2+^-free intracellular medium (ICM; 10 mM NaCl, 120 mM KCl, 1 mM KH_2_PO_4_, 20 mM HEPES-Tris, pH 7.2), supplemented with 2 μM thapsigargin to block the SERCA pump and 15 mM succinate. 1μM Fura-FF was added to the cell suspension for the detection of free bath Ca^2+^. Fura-FF fluorescence was monitored in a multiwavelength excitation dual-wavelength emission spectrofluorometer (Delta RAM, PTI, HORIBA). Bath Ca^2+^ is shown as the [Ca^2+^]_out_ (μM). Ca^2+^ boluses (10μM) were added until the mitochondria stopped clearing the Ca^2+^ bolus, and then the mitochondrial uncoupler, FCCP (3 μM), was added to confirm mitochondrial depolarization. All Ca^2+^ flux measurements were performed at 37°C with constant stirring.

#### Mitochondrial fractionation for CCDC58 localization

HeLa, HEK293T, and COS-7 cells were cultured in 150 mm^2^ cell culture dishes and transfected with CCDC58-Flag using the FuGENE HD transfection reagent per manufacturer instructions. Mitochondria were isolated by utilizing the mitochondria isolation kit for cultured cells (Abcam Cat# ab110170), as per manufacturer instructions. Total cell lysate (TCL) and cytosol (cyto) were collected, and the mitochondrial pellet was permeabilized with digitonin (0.2 mg/mL). After permeabilization, mitochondrial pellet and supernatant (sup) is collected. Trypsinization (Trypsin 0.1% w/v) of intact and permeabilized mitochondria was performed to validate the CCDC58 localization in mitochondrial compartments. Mitochondrial compartment-specific marker antibodies, TOM20 for outer mitochondrial membrane, Cytochrome c for intermembrane space, ATP5A for inner mitochondrial membrane, and PPIF for mitochondrial matrix were used.

#### Evaluation of intracellular SARS-CoV-2 protein localization and colocalization with ER and mitochondria using live-cell confocal imaging system

hiPSC derived cardiomyocytes or COS-7 cells transiently transfected with the mRFP-tagged SARS-CoV-2 plasmid constructs were grown on 25-mm 0.1% gelatin or collagen coated glass coverslips, respectively. 48 h post-transfection, cells were loaded with 1 μM of ER Tracker blue-white DPX (ex/em 374/430 nm) and mitochondrial marker Dihydrorhodamine (2.5 μM; ex/em 505/524 nm) in cell growth media for 30 min at 37°C (5% CO2). The cells were washed and imaged within a temperature-controlled environmental chamber set at 37°C using a Leica TCS SP8 live-cell confocal imaging system. The subcellular localization of the cells transfected with SARS-CoV-2 proteins was imaged using a 100x oil objective and analyzed using Leica Application Suite X. The colocalization of Cox-8a mRFP and the SARS-CoV-2 proteins (Vector control, M Protein, Orf9c, Orf3a, Nsp6, Nsp10) with the ER (ER BFP) and mitochondria (DHR 123) were quantitatively measured using the Leica Application Suite X. The levels of colocalization were plotted by calculating the Pearson's co-efficient of colocalization and overlap co-efficient and the % of colocalization was determined. The values were plotted using GraphPad Prism version 8.

#### Evaluation of mitochondrial phenotype

The mitochondrial phenotypic changes in cells infected with SARS-CoV-2 virus was evaluated using genetically encoded mitochondrial sensors and mitochondria staining dyes. hiPSC derived cardiomyocytes were grown on 25-mm gelatin-coated glass coverslips and were infected with Ad-mito-GCamp6 (ex/em 480/510 nm: MOI 5). After 48 h, the cells were infected with SARS-CoV-2 virus (MOI 1) for 24 h and fixed with 10% Neutral buffered formalin. The fixed cells were imaged on a Leica TCS SP8 confocal imaging system using a 100x oil objective. The levels of mitophagy and mitochondrial phenotype was imaged, and the mitochondrial length, area and perimeter were measured using the Leica Application Suite X. The results were tabulated and plotted using GraphPad Prism version 8.

hiPSC derived cardiomyocytes were grown on 25-mm gelatin-coated glass coverslips and were infected with SARS-CoV-2 virus (MOI 1) for 24 h and were stained with Mitotracker deep red FM (ex/em 644/665 nm) for 30 min. The stained cells were fixed with 10% Neutral buffered formalin. The fixed cells were imaged on a Leica TCS SP8 confocal imaging system using a 100x oil objective. The levels of mitophagy mitochondrial phenotype i.e., mitochondrial length, area and perimeter were measured using the Leica Application Suite X. The results were tabulated and plotted using GraphPad Prism version 8.

#### Visualization of autophagy using confocal microscope

To evaluate the suicidal effect of cells in response to the SARS-CoV-2 infection, hiPSC derived cardiomyocytes were infected with LC3 GFP/RFP tandem adenovirus (GFP ex/em 488/510 nm; RFP ex/em 552/584 nm; MOI 5). After 24 h, the cells were infected with SARS-CoV-2 (MOI 1) for 24 h and fixed with 10% neutral buffered formalin. The fixed cells were imaged on a Leica TCS SP8 imaging system using a 100x oil objective. The number of vesicular puncta were counted and plotted using GraphPad Prism version 8.

#### Assessment of SARS-CoV-2 protein localization

To visualize the ectopic localization of the SARS-CoV-2 viral proteins, hiPSC derived cardiomyocytes were infected with adenoviruses encoding tubulin-GFP (ex/em 484/507 nm; MOI 5) and Sec61 mCherry (Vector BioLabs, ex/em 587/610 nm; MOI 5). After 24 h, the cells were infected with SARS-CoV-2 virus (MOI 1) for 24 h and fixed with 10% neutral buffered formalin and imaged on a Leica TCS SP8 imaging system using a 100X oil objective. The phenotypic implications of the SARS-CoV-2 were analyzed using the Line Scan analysis, Leica Application Suite X.

#### Measurement of mitochondrial Ca^2+^ uptake in a permeabilized cell system

HEK293T cells stably expressing negative shRNA, CCDC58 KD, or CCDC58-pBSD; as well as CCDC58 KD HEK293T cells stably expressing SARS-CoV-2 proteins (M Protein, Orf9c, Orf3a, Nsp6, Nsp7) or only expression vector, were used for the measurement of mitochondrial Ca^2+^ uptake. Briefly, the cells were washed with Ca^2+^ and Mg^2+^ free DPBS, pH 7.4. An equal number of cells (∼4-5x10^6^ cells) were resuspended and permeabilized with 40 μg/mL digitonin in 1.5 mL ICM. Mitochondrial calcium uptake was measured in the presence of 1.5 mM Mg^2+^/ATP and 5 mM succinate, with or without 5 μM cyclosporin A (CsA). The permeabilized cells were loaded with Fura-2 FF (1 μM; ex 340/380 nm and em 510 nm). The measurement of extramitochondrial Ca^2+^ clearance was used as an indicator of [Ca^2+^]_m_ uptake. Fluorescence was measured in a multi-wavelength excitation and dual-wavelength emission spectrofluorometer (Delta RAM, PTI, HORIBA). JC-1 (800 nM) was used simultaneous measurement of mitochondrial membrane potential. [Ca^2+^]_out_ is represented as the excitation ratio (ex/em 340/380 nm) of FURA2-FF/FA fluorescence and ΔΨ_m_ as the ratio of fluorescence of J-aggregate (ex/em 570/595 nm) and monomer (ex/em 490/535 nm) forms of JC-1. Multiple Ca^2+^ pulses (10 μM) and FCCP (10 μM) were added at the indicated time points. All the experiments were performed at 37°C with constant stirring (Mallilankaraman et al., 2012b; Tomar et al., 2019).

#### Mitochondrial oxygen consumption rate

COS-7 cells stably expressing the vector or the SARS-CoV-2 plasmid constructs (M Protein, Orf9c, Orf3a, Nsp6, Nsp7) as well as Neg shRNA and CCDC58 KD HEK293T cells were plated on 96 well Agilent Seahorse XF Cell Culture Microplates. The cells were plated at a density of 4 × 10^4^ cells per well. Cells were cultured in the normal growth media overnight. Media was changed to Seahorse XF Cell Mito Stress Test Kit (Agilent) assay media supplemented with glucose, glutamine, pyruvate concentrations equivalent to that of the growth media 1 h before the experiment start time. After media replacement, cells were placed in a CO_2_-free incubator for 1 h. Oxygen consumption rate (OCR) and extracellular acidification rate (ECAR) was measured at 37°C in an XF96 extracellular flux analyzer (Seahorse Bioscience, Agilent), which had been previously calibrated using Seahorse XF Calibrant solution (Seahorse Bioscience, Agilent) in a CO_2_-free incubator overnight. Respiratory chain inhibitors were then loaded into the XF96 flux analyzer, and during the run, added sequentially to cells at indicated time points. The cells received oligomycin, FCCP, and a mixture of antimycin A and rotenone at concentrations of 2 μM, 2 μM, and 0.5 μM, respectively. Data was collected using Agilent Seahorse Wave 2.6.1 Desktop software and exported to GraphPad Prism version 8 for analysis (Nemani et al., 2018, 2020).

#### Measurement of spontaneous cytoplasmic Ca^2+^ cycling

Human iPSC-CMs were grown on 0.1% gelatin-coated 25 mm glass coverslips. SARS-CoV-2 protein expressing hiPSC-CMs were loaded with Fluo-4/AM (5 μM, 30 min) in extracellular media at room temperature. Coverslips were mounted in a temperature controlled microincubator and imaged (Mallilankaraman et al., 2012b; Shanmughapriya et al., 2018). Spontaneous Ca^2+^ cycling was recorded every 3 seconds (TCS SP8 live cell imaging system, Leica, Wetzlar, Germany) at 488 nm excitations using a 100X oil objective. Images were analyzed and quantified by Leica application Suite X.

#### L-type Ca^2+^ current density recording

L-type (*I*_*Ca-L*_) calcium channel current was measured using whole-cell patch-clamp technique at room temperature. The whole-cell membrane currents were recorded using a low noise patch-clamp amplifier (Axopatch 200B; Axon Instruments, Hawthorn, Victoria, Australia) interfaced via a Digidata 1550B (Axon Instruments) to a PC running the pClamp 11 suite of software (Axon Instruments). All currents were filtered at 1 kHz. The current-voltage curve was generated by voltage-clamp protocols consisting holding potential of −80 mV followed by a 3s long pre-pulse at −50 mV to inactivate Na^+^ and T-type Ca^2+^ channels, then a family of 300 ms depolarization from −50 mV to +60 mV in 10 mV increments. Patch pipettes were pulled from borosilicate glass capillaries (World Precision Instruments, Inc., Sarasota, Florida) using a model P-97 Flaming-Brown micropipette puller (Sutter Instrument Company, Novato, CA) and fire-polished on a micro-forge (MF-830; Narishige Scientific Instrument, Tokyo, Japan) to have a 3–5 MΩ when backfilled with the pipette (intracellular) solution. The pipette solution comprised (mM): CsCl (120), MgCl_2_ (3), EGTA (10), Mg^2+^/ATP (5), HEPES (5); pH was adjusted to 7.2 with CsOH. The bath (extracellular) solution comprised (mM): NaCl (14), CsCl (10), CaCl_2_ (1.8), MgCl_2_ (1), HEPES (10), 10 Glucose (10) and tetrodotoxin (0.01, TTX, TOCRIS, USA) to block Na^+^ currents; pH was adjusted to 7.4 with NaOH. *I*_*Ca-L*_ was confirmed by its sensitivity to 10 μM of nifedipine. All chemicals were purchased from Sigma (Sigma-Aldrich, St. Louis, MO) unless noted otherwise.

#### Assessment of mitochondrial reactive oxygen species

To study the levels of mitochondrial ROS, Neg shRNA and CCDC58 KD HEK293T cells were grown on 25 mm collagen-coated glass coverslips overnight and loaded with the mitochondrial superoxide sensitive fluorophore MitoSOX red (ex/em 556/610 nm, 5 μM) for 30 min at 37°C. The stained cells were washed and imaged using a Leica SP8 confocal microscope, coupled with a temperature-controlled environmental chamber. The images were acquired and analyzed using the Zeiss LSM510 Meta NLO using ZEN2010 software (Mukopadhyay et al., 2007).

#### Calcein fluorescence measurement

HeLa neg shRNA and CCDC58 KD cells were treated with ionomycin (25 μM) for 6 h ± CsA (5 μM) or t-BH (100 μM) for 6 h ± CsA (1 μM) and stained with PTP opening indicator calcein-AM. The reduction in fluorescence following treatments was assessed by confocal microscopy on comparison with the untreated controls (Shanmughapriya et al., 2015a).

#### Total cellular ATP levels

Cellular ATP levels were measured using CellTiter-Glo luminescent kit, and luminescence intensity was detected using the Infinite M1000 PRO plate reader (Tecan).

#### SARS-CoV-2 protein expression and Western analysis

hiPSC-CMs were transiently transfected with mRFP/Flag-tagged SARS-CoV-2 plasmids construct individually. 48 h post-transfection, cell lysates were prepared for Western blot analysis and SARS-CoV-2 viral protein expression was probed using FLAG antibody.

#### Immunoprecipitation

For immunoprecipitation, cell extracts were prepared using IP lysis buffer (ThermoFisher; 87788) containing protease and phosphatase inhibitor cocktail (ThermoFisher 78440). To assess the interaction of SARS-CoV-2 proteins with PPIF, SPG7 or CCDC58, FLAG-tagged SARS-CoV-2 plasmid constructs were co-transfected with HA-tagged PPIF or HA-tagged SPG7 or Myc-tagged CCDC58 plasmid in COS7 cells. Cell lysates were immunoprecipitated with HA for PPIF or SPG7 and with Myc for CCDC58. To determine the protein-protein interaction between SARS-CoV-2 proteins and PTP complex components, immunoprecipitated samples were probed with anti-FLAG antibody. To confirm the PPIF or SPG7 interaction with SARS-CoV-2 proteins, reverse immunoprecipitation was performed using anti-FLAG antibody and immunoprecipitated samples were probed with anti-HA antibody. To assess the CCDC58 binding with PPIF or SPG7, co-transfected lysates were immunoprecipitated with Myc. The whole-cell lysate and the eluate were probed with anti-Myc or anti-HA antibodies, respectively. To assess the interaction between SARS-CoV-2 proteins and endogenous SPG7 or ANT or ATP Synthase, FLAG-tagged SARS-CoV-2 plasmids transfected cell lysates were immunoprecipitated with anti-FLAG antibody and eluates were probed with either endogenous SPG7 or or ANT or ATP Synthase.

#### MTT assay

iPSC-CMs were plated on 0.1% gelatin coated 96-well plate. Cells were treated with cyclosporin-A (1 μM) prior to SARS-CoV-2 infection with 50 PFU for 24 h. The cell viability was determined using the MTT (3-[4,5-dimethylthiazol-2-yl]-2,5- diphenyl-tetrazolium bromide) assay according to the manufacturer protocol.

### Quantification and statistical analysis

#### Statistical analysis

All data are expressed as mean ± SEM unless otherwise indicated. Statistical significance was evaluated using a Student's unpaired *t*-test for one or multiple samples, and one-way ANOVA followed by Tukey's multiple comparison test as needed with p < 0.05 considered to be statistically significant. Data from cells was obtained using built-in Leica software and were analyzed and plotted using GraphPad Prism version 8. All other data were analyzed using Microsoft Excel 2016, GraphPad Prism version 8. Charts and figures were created using GraphPad Prism version 8 software, Microsoft Office 365 Word, Microsoft Office 365 Excel, and Canvas 11 software.

## Data Availability

• All data are included in the published article and the supplemental information files or are available from the lead contact upon request. • This paper does not report original code. • Any additional information required to reanalyze the data reported in this paper is available from the lead contact upon request.

## Supplemental information

Document S1. Figures S1–S6 and Table S1

Data S1. RNA-seq analysis of PBMCs derived from SARS-CoV-2 infected patients with varying degrees of severity and uninfected human subjects, related to Figures 1, 3, and 5 In excel files, the “A” refers to uninfected subjects. The “B” refers to mild symptomatic SARS-CoV-2-infected patients. The “C” refers to severely affected SARS-CoV-2 patients

Data S2. RNA-seq analysis of iPSCs-derived cardiomyocytes infected with SARS-CoV-2 virus, related to Figure 7 Supplemental files include differential splice variant expression data of control (M1, M2, and M3) and SARS-CoV-2-infected iPSCs (S1, S2, and S3)

Data S3. RNA-seq analysis of iPSCs-derived cardiomyocytes infected with SARS-CoV-2 virus, related to Figure 7 Supplemental files include GO, hit counts, QC, and stats of control (M1, M2, and M3) and SARS-CoV-2-infected iPSCs (S1, S2, and S3)
