# Supplementary material for: SARS-CoV-2 infection enhances mitochondrial PTP complex activity to perturb cardiac energetics
Source: iScience. 2022 Jan 1;25(1):103722. doi: 10.1016/j.isci.2021.103722 (PMC8720045; doi:10.1016/j.isci.2021.103722)
Supplement: Document S1. Figures S1–S6 and Table S1 [file mmc1.pdf]

iScience, Volume *25*

Supplemental information

SARS-CoV-2 infection enhances mitochondrial PTP complex activity to perturb cardiac energetics

Karthik Ramachandran, Soumya Maity, Alagar R. Muthukumar, Soundarya Kandala, Dhanendra Tomar, Tarek Mohamed Abd El-Aziz, Cristel Allen, Yuyang Sun, Manigandan Venkatesan, Travis R. Madaris, Kevin Chiem, Rachel Truitt, Neelanjan Vishnu, Gregory Aune, Allen Anderson, Luis Martinez, Wenli Yang, James D. Stockand, Brij B. Singh, Subramanya Srikantan, W. Brian Reeves, and Muniswamy Madesh

Table S1: Patient characteristics and blood test results. Related to Figure 1

**Patient group**	**Normal**	**Normal**	**Mild/Moderate**	**Mild/Moderate**	**Mild/Moderate**	**Severe**	**Severe**
Patient ID#	Normal-24C	Normal-25C	Symptomatic-11B	Symptomatic-12B	Symptomatic-15B	Symptomatic-3A	Symptomatic-4A
Age	52	59	56	85	69	69	57
Gender	F	F	F	F	F	M	M
Chronic/Preexisting condition	-	-	NIDDM-2, HLD, Morbid Obesity, HTN, CKD, BPD, Sleep Apnea, Schizophrenia	NIDDM-2, HLD, CHF, EF, HTN, CKD Aortic valve replacement	HTN, HLD, CHF, AICD, Volume overload	HLD	HCV, Obesity, HTN, HLD, ILD, Lung and Liver transplant
Self-reported initial symptoms	-	-	Fever, Cough, Myalgia, Anorexia, thick phlegm	Cough, Malaise, Diarrhea	Cough, Myalgia, SOB	Fever, Cough, Diarrhea	Worsening SOB, ARDS
Days from symptom onset to PCR test	-	-	3	3	6	5	5
PCR result	-	-	positive	positive	positive	positive	positive
SARS-CoV-2 IgG	Negative	Negative	Negative	Negative	Negative	positive	positive
Patient Isolation	-	-	At home quarantine	Hospitalized-COVID ward	At home quarantine	Hospitalized –COVID ward to ICU	Hospitalized - ICU
ICU/Intubation	-	-	No	No	No	Yes/Yes	Yes/Yes
Outcome	-	-	Recovered	Recovered & Discharged	Recovered	Deceased	Recovered & Discharged
LOS	-	-	-	7 days	-	29 days	77 days
CRP (<= 5 mg/L)				130.9		149	20
Hs-cTnT (22 ng/L)	<6	<6	15	80	12	17	129
NT-proBNP (<900 pg/ml)	35	21	245	1456 (<1800)	756	2475	8014
WBC (4-11 x 10^9/L)	-	-	4.59	8.30	3.44	9.23	25.42
Platelets (150-450 x 10^9/L)	-	-	265	207	155	211	158

A

Figure S1

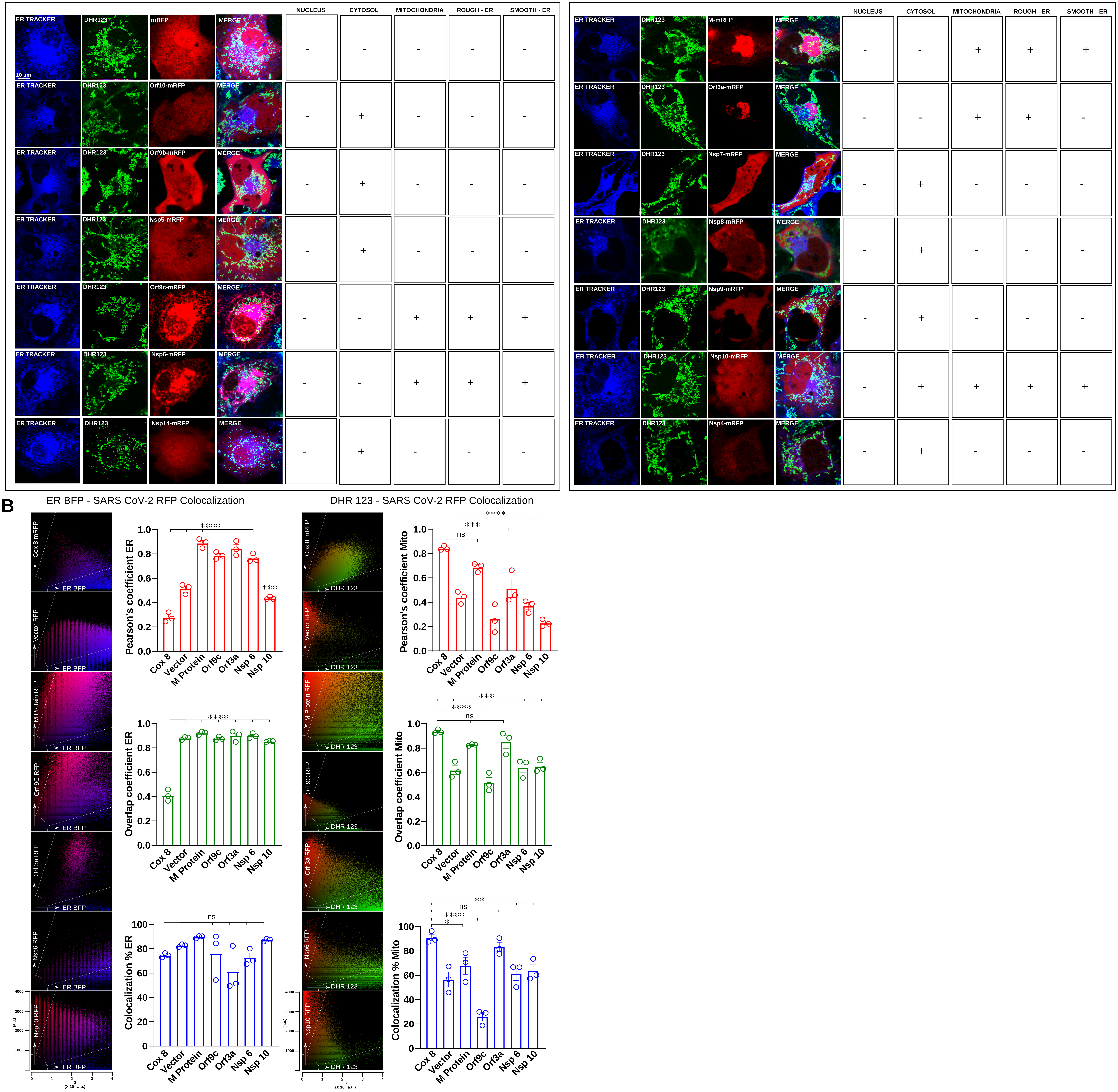

ER TRACKER
DHR123
mRFP
MERGE
10 µm
NUCLEUS
CYTOSOL
MITOCHONDRIA
ROUGH - ER
SMOOTH - ER
Orf10-mRFP
Orf9b-mRFP
Nsp5-mRFP
Orf9c-mRFP
Nsp6-mRFP
Nsp14-mRFP
M-mRFP
Orf3a-mRFP
Nsp7-mRFP
Nsp8-mRFP
Nsp9-mRFP
Nsp10-mRFP
Nsp4-mRFP
B
ER BFP - SARS CoV-2 RFP Colocalization
DHR 123 - SARS CoV-2 RFP Colocalization
Cox 8 mRFP
Vector RFP
M Protein RFP
Orf 9C RFP
Orf 3a RFP
Nsp6 RFP
Nsp10 RFP
ER BFP
DHR 123
(a.u.)
(X 10^3 a.u.)
Pearson's coefficient ER
Overlap coefficient ER
Colocalization % ER
Pearson's coefficient Mito
Overlap coefficient Mito
Colocalization % Mito
Cox 8
Vector
M Protein
Orf9c
Orf3a
Nsp 6
Nsp 10
****
***
**
*
ns

Figure S1: Subcellular distribution of Sars-Cov-2 proteins in Cos-7 cells, Related to Figure 2.

(A). Confocal images of COS-7 cells expressing mRFP-tagged SARS-CoV-2 plasmids constructs (Vector, NSP4, 5, 6, 7, 8, 9, 10, and 14, M Protein, ORF3a, ORF9b, ORF9c, and ORF10). 48 hours post transfection, cells were loaded ER tracker, and mitochondrial indicator (DHR123) and confocal live cell images were acquired. n=3 independent experiments.

(B). Colocalization analysis of SARS-CoV-2 proteins in COS-7 cells. n=3 independent experiments.

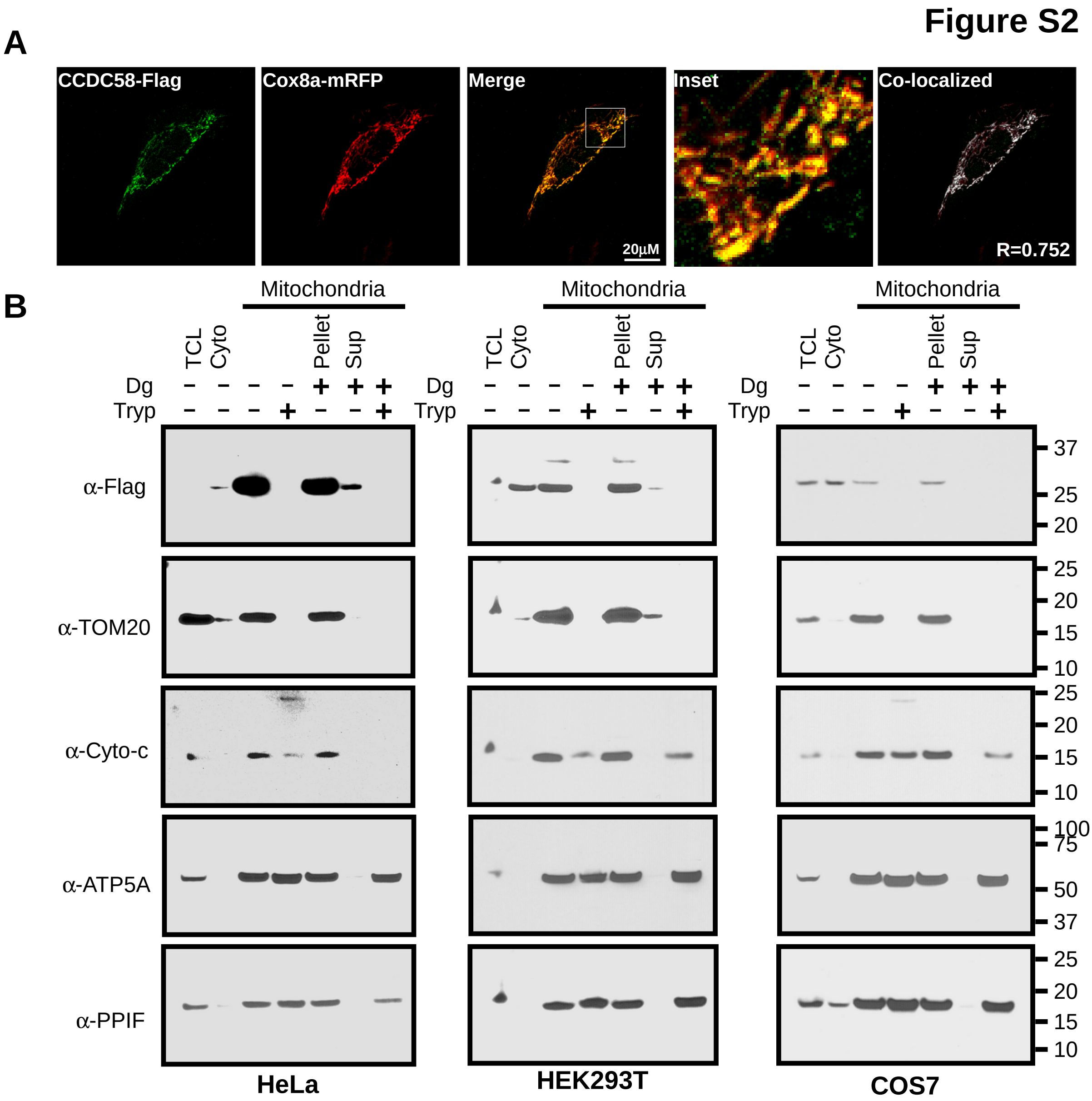

Figure S2
A
CCDC58-Flag
Cox8a-mRFP
Merge
Inset
Co-localized
20µM
R=0.752
B
Mitochondria
TCL
Cyto
Pellet
Sup
Dg
Tryp
α-Flag
α-TOM20
α-Cyto-c
α-ATP5A
α-PPIF
37
25
20
15
10
100
75
50
HeLa
HEK293T
COS7

Figure S2: Assessment of cellular and subcellular localization of CCDC58 in multiple cell types, Related to Figure 3

(A) HeLa cells were transiently cotransfected with FLAG-tagged CCDC58 and mitochondrial marker COX8A-mRFP plasmid constructs. Immunofluorescence analysis of CCDC58 localization shows the mitochondrial localization.

(B) HeLa, HEK293T or COS-7 cells stably expressing FLAG-tagged CCDC58 were subjected to subcellular fractionation. CCDC58 distribution was were assessed by Western blotting using appropriate protein markers.

Figure S3

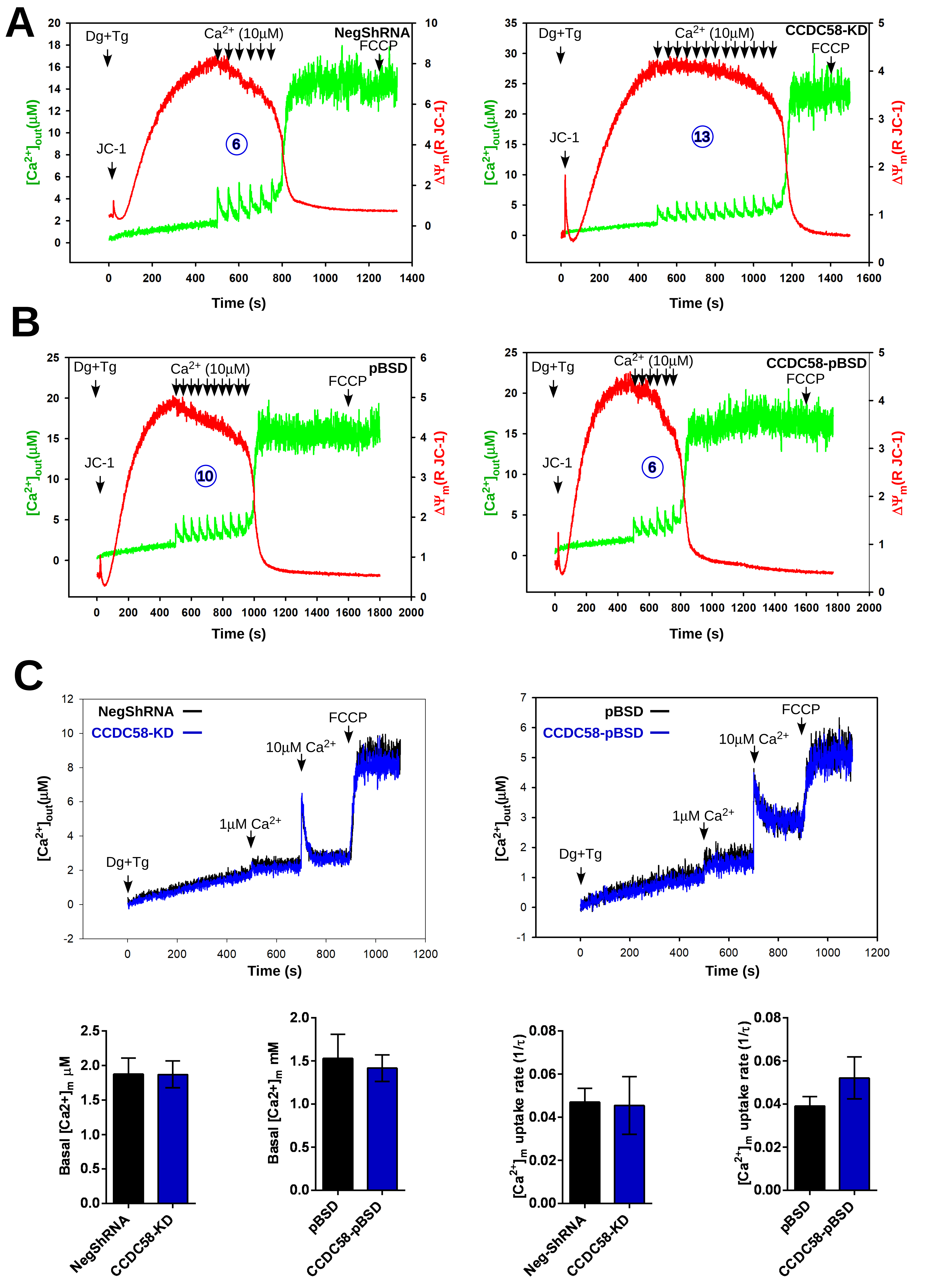

A
Dg+Tg
JC-1
Ca2+ (10μM)
NegShRNA
FCCP
6
[Ca2+]out(μM)
ΔΨm(R JC-1)
Time (s)
CCDC58-KD
13
B
pBSD
10
CCDC58-pBSD
C
NegShRNA
CCDC58-KD
10μM Ca2+
1μM Ca2+
pBSD
CCDC58-pBSD
Basal [Ca2+]m μM
Basal [Ca2+]m mM
[Ca2+]m uptake rate (1/τ)
Neg-ShRNA

Figure S3: Knockdown of CCDC58 enhances mitochondrial CRC without altering MCU-mediated Ca^{2+} uptake, Related to Figure 3.
(A-B) (A) Control, CCDC58 KD, (B) vector alone (pBSD) or CCDC58 overexpressing HeLa cells were permeabilized and mitochondrial CRC was measured.
(C) Control, CCDC58 KD, vector alone or CCDC58 overexpressing HeLa cells were permeabilized and basal and MCU activity rate were calculated.

Figure S4

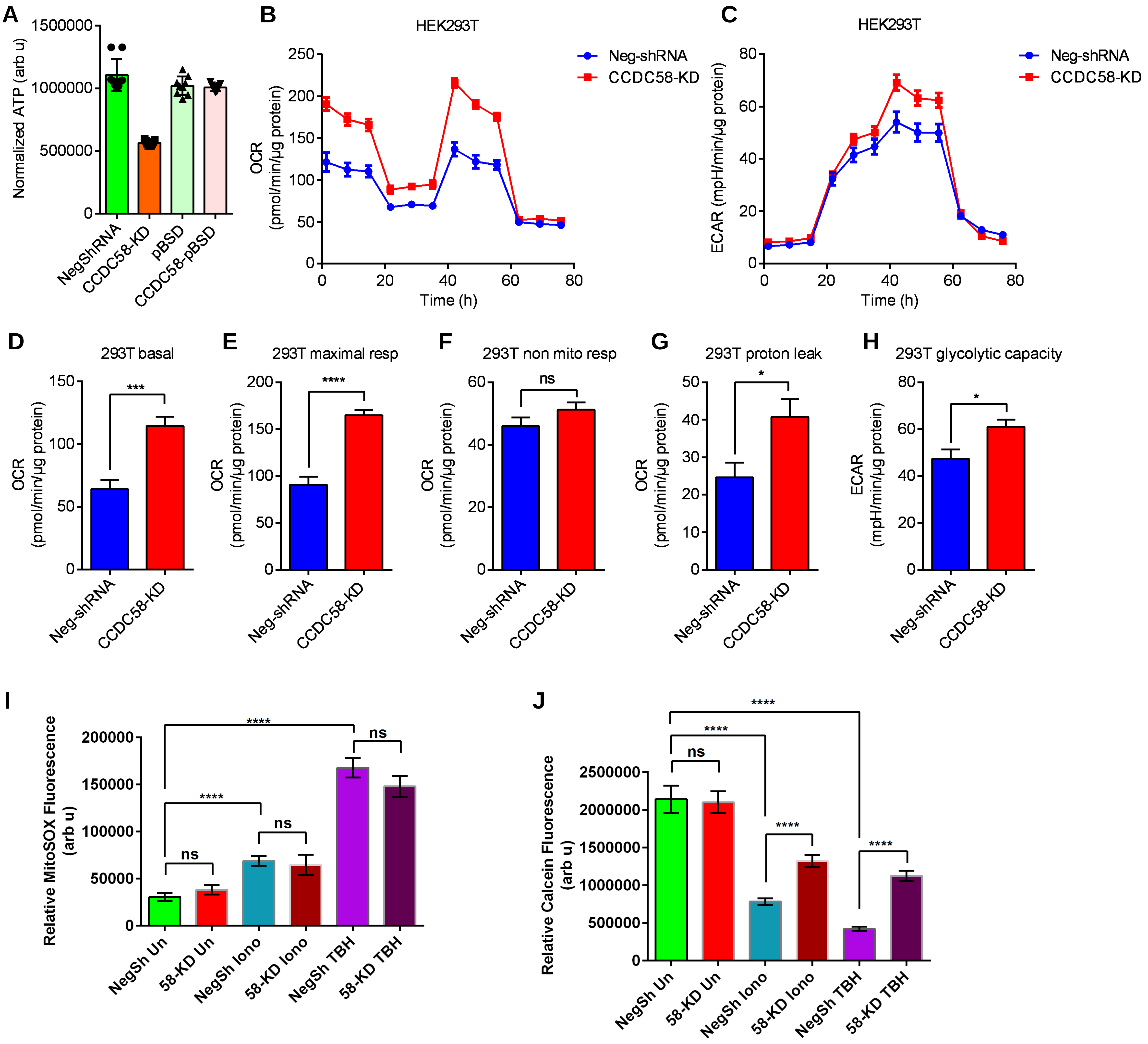
A
Normalized ATP (arb u)
NegShRNA
CCDC58-KD
pBSD
CCDC58-pBSD
B
HEK293T
OCR (pmol/min/µg protein)
Time (h)
Neg-shRNA
CCDC58-KD
C
HEK293T
ECAR (mpH/min/µg protein)
Time (h)
Neg-shRNA
CCDC58-KD
D
293T basal
OCR (pmol/min/µg protein)
***
E
293T maximal resp
OCR (pmol/min/µg protein)
****
F
293T non mito resp
OCR (pmol/min/µg protein)
ns
G
293T proton leak
OCR (pmol/min/µg protein)
*
H
293T glycolytic capacity
ECAR (mpH/min/µg protein)
*
Neg-shRNA
CCDC58-KD
I
Relative MitoSOX Fluorescence (arb u)
****
ns
NegSh Un
58-KD Un
NegSh Iono
58-KD Iono
NegSh TBH
58-KD TBH
J
Relative Calcein Fluorescence (arb u)
****
ns
NegSh Un
58-KD Un
NegSh Iono
58-KD Iono
NegSh TBH
58-KD TBH

Figure S4: Assessment of cellular ATP and mitochondrial OCR, mitochondrial ROS production, and calcein retention of CCDC58 KD cells following exposure to stress conditions, Related to Figure 3.

(A) Measurement of cellular ATP levels. Mean $\pm$ SEM, n = 5-7 independent experiments.

(B and C) Measurement of OCR and ECAR of CCDC58 KD cells. Mean $\pm$ SEM, n = 5-7 replicates.

(D-H) Analysis of basal, maximal, non-mito OCR, proton leak, and glycolytic capacity. Data are presented as the mean $\pm$ SEM, n = 3-4 independent experiments. *$p < 0.05$, ***$p < 0.001$, ****$p < 0.0001$, n.s., not significant.

(I) Measurement of mitochondrial MitoSOX red fluorescence with or without ionomycin and t-BH challenge for 60 min. Data are presented as the mean $\pm$ SEM, n = 3-5 independent experiments. ****$P < 0.0001$, n.s., not significant.

(J) Measurement of Calcein retention following ionomycin or t-BH challenge for 4 hours. Data are presented as the mean $\pm$ SEM, n = 3-4 independent experiments. ****$P < 0.0001$, n.s., not significant.

Figure S5

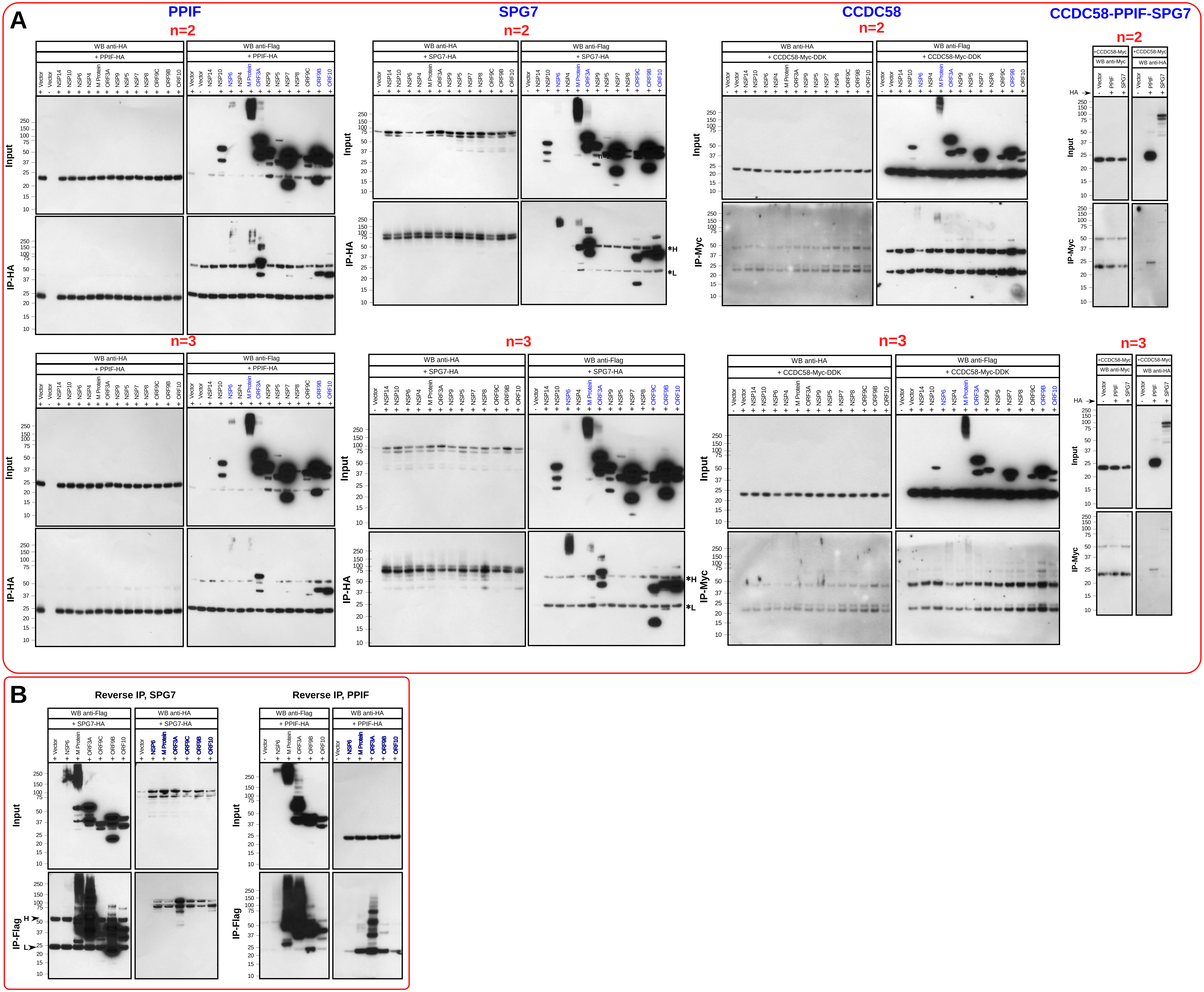

A
PPIF
SPG7
CCDC58
CCDC58-PPIF-SPG7
n=2
n=3
WB anti-HA
WB anti-Flag
WB anti-Myc
+ PPIF-HA
+ SPG7-HA
+ CCDC58-Myc-DDK
+CCDC58-Myc
Vector
NSP14
NSP10
NSP6
NSP4
M Protein
ORF3A
NSP9
NSP5
NSP7
NSP8
ORF9C
ORF9B
ORF10
PPIF
SPG7
Input
IP-HA
IP-Myc
*H
*L
HA
B
Reverse IP, SPG7
Reverse IP, PPIF
IP-Flag
H
L

Figure S5. SARS-CoV-2 proteins interact with mitochondrial PTP complex, Related to Figure 4.

(A) COS-7 cells were cotransfected with HA-tagged PPIF and FLAG-tagged SARS-CoV-2 protein plasmid constructs. Following immunoprecipitation with HA antibody, total cell lysates and immunoprecipitated materials were subjected to Western blot analysis. Cell lysates were probed with anti-FLAG or anti-HA antibodies. Immunoprecipitated samples were probed with anti-FLAG (top right) and anti-HA antibodies (bottom right). Western blot analysis of cell lysates (left) or immunoprecipitates (right) from COS-7 cells coexpressing HA-tagged SPG7 and FLAG-tagged SARS-CoV-2 protein plasmid constructs. Western blot analysis of cell lysates (left) or immunoprecipitates (right) from COS-7 cells coexpressing Myc-tagged CCDC58 and FLAG-tagged SARS-CoV-2 protein plasmid constructs. n = 3.

(B) Reverse IP analysis of (A) for SARS-CoV-2 proteins and Mitochondrial PTP Complex interactions.

Figure S6

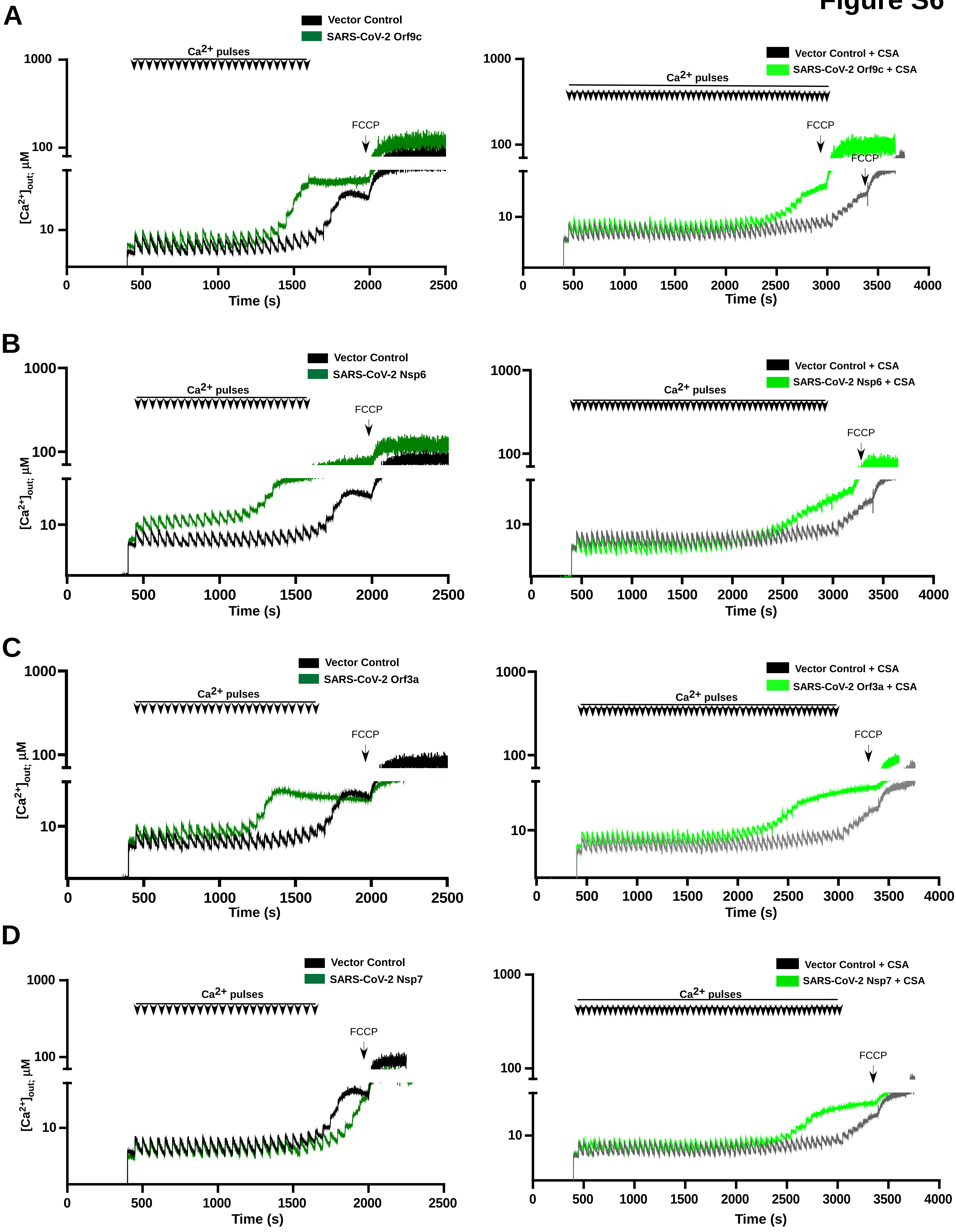
A
Vector Control
SARS-CoV-2 Orf9c
Ca2+ pulses
FCCP
[Ca2+]out; μM
Time (s)
Vector Control + CSA
SARS-CoV-2 Orf9c + CSA
B
Vector Control
SARS-CoV-2 Nsp6
Vector Control + CSA
SARS-CoV-2 Nsp6 + CSA
C
Vector Control
SARS-CoV-2 Orf3a
Vector Control + CSA
SARS-CoV-2 Orf3a + CSA
D
Vector Control
SARS-CoV-2 Nsp7
Vector Control + CSA
SARS-CoV-2 Nsp7 + CSA

Figure S6: Effect of cyclosporin A on SARS-CoV-2 protein-induced mitochondrial CRC change, Related to Figure 6.
(A-D) Representative traces of number of Ca^{2+} pulses cleared by mitochondria (CRC). Vector or SARS-CoV-2 ORF9c, NSP6, ORF3a, and NSP7 stably expressing HEK293 cells were permeabilized and exposed to boluses of 10 μM Ca^{2+} pulses with (C) or without (D) cyclosporin A (1 μM) at the indicated time point. n=3-5 independent experiments.
