# Supplementary material for: SARS-CoV-2 infection enhances mitochondrial PTP complex activity to perturb cardiac energetics
Source: iScience. 2022 Jan 1;25(1):103722. doi: 10.1016/j.isci.2021.103722 (PMC8720045; doi:10.1016/j.isci.2021.103722)
Supplement: Data S3. RNA-seq analysis of iPSCs-derived cardiomyocytes infected with SARS-CoV-2 virus, related to Figure 7 — Supplemental files include GO, hit counts, QC, and stats of control (M1, M2, and M3) and SARS-CoV-2-infected iPSCs (S1, S2, and S3) [file mmc4.zip › Report/QC-Report/M3_QC_report.html]

GENEWIZ NGS Sample QC Report


- Sample Information
- Sample Sequencing QC Summary

# GENEWIZ NGS Sample QC Report

## 1. Sample Information

|  |  |
| --- | --- |
| Customer | subramanya srikantan |
| Email | subramanyasr@uthscsa.edu |
| Quote Number | 30-384610326 |
| Configuration | HiSeq 2x150 PE HO HiSeq 2x150bp |
| Sample | M3 |

## 2. Sample Sequencing QC Summary

Raw data quality was evaluated with FastQC.

### 2.1 Per base quality score distribution

Figure 2.1.1 shows an overview of the range of quality scores at each base across all the reads. The x-axis represents the position in the read. The y-axis represents the quality score.

Figure 2.1.1 Base quality score distribution

### 2.2.1 Reads quality score distribution

Figure 2.2 shows the percentage of reads that have a given quality score. The x-axis represents the quality score. The y-axis represents the percentage of reads that have an equal or higher quality score.

Figure 2.2.1 Reads quality score distribution

### 2.3.1 Reads GC content distribution

Figure 2.3 shows the distribution of average GC content across all the reads. The x-axis represents the GC content percentage. The y-axis represents the percentage of reads with the given GC content.

Figure 2.3.1 Reads GC content distribution

- GENEWIZ Next Generation Sequencing
- Email: ngs@genewiz.com
- Phone: 908-222-0711 ext 1
